# Buckling of elastomer-embedded thermoplastic microspheres under uniaxial compression revealed by in-situ X-ray micro-CT

**DOI:** 10.1038/s43246-026-01214-w

**Published:** 2026-06-05

**Authors:** Matthew E. Curd, Gareth Wyn Jones, Zeshan Yousaf, Steve Edmondson, Parmesh Gajjar, Michael J. A. Smith, Partha P. Paul, Rhys Thomas, Eleanor Russell, Tom Shearer, Thomas White, Jonathan Perrin, Timm Weitkamp, Mario Scheel, Guillaume Daniel, William J. Parnell

**Affiliations:** 1 Department of Mathematics, University of Manchester, Manchester, UK; 2 Henry Royce Institute, Department of Materials, University of Manchester, Manchester, UK; 3 ESRF, Grenoble, France; 4 Synchrotron SOLEIL, Saint-Aubin, France

**Keywords:** Polymers, Composites, Metamaterials

## Abstract

Understanding and predicting the morphological changes of filler particles within composites under load is critical to design improved materials. Syntactic foams, consisting of microspheres embedded in a solid matrix, have recently emerged as an ultra-lightweight metamaterial with highly tuneable mechanical properties. Glass microspheres are commonly used in syntactic foams, but can fracture, leading to a loss in the bulk mechanical properties. It is thought that hollow thermoplastic microspheres instead buckle, inducing strong, reversible, constitutive nonlinearity in the syntactic foam. Here we demonstrate, in-situ, the buckling of embedded thermoplastic shells under uniaxial compression. The results validate several important predictions from theory; regardless of sphere size, equatorial buckling prevails whenever the shell walls are thin relative to their diameter. Furthermore, the buckling wavelength is shown to be tuneable via different mechanical and geometrical parameters for the microspheres and/or matrix. Now better understood, we anticipate that tuneable, controlled and coordinated microsphere buckling can be exploited in diverse applications including strain sensing.

## Abstract (web summary)

Glass microspheres are commonly used in syntactic foams but can fracture, leading to a loss in the bulk mechanical properties. Here we demonstrate in-situ the buckling of embedded thermoplastic shells under uniaxial compression.

## Introduction

**Fig. 1:**
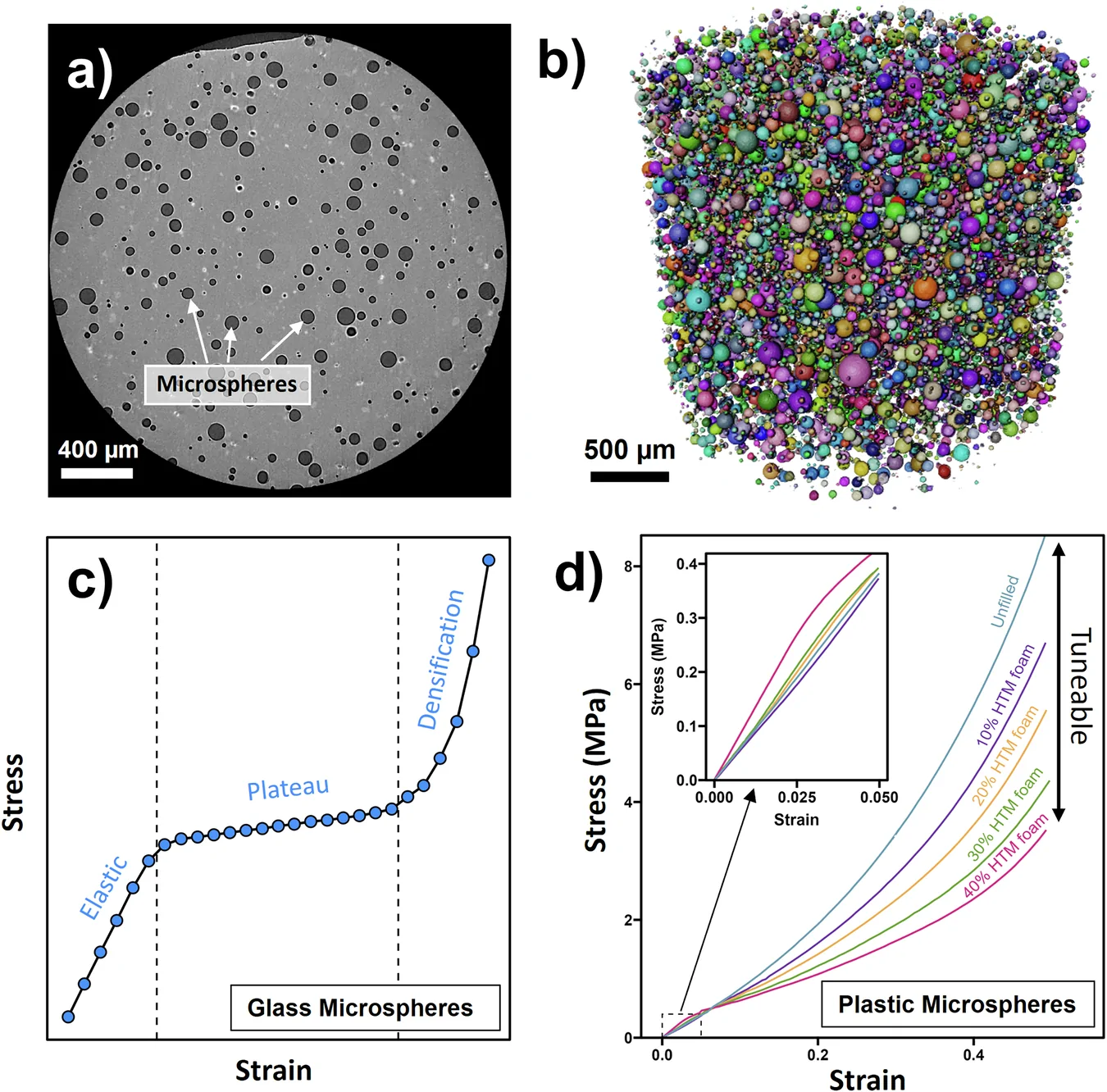
Depictions of the microstructure and mechanical performance of a syntactic foam. **a** X-ray CT cross-section showing a polyurethane-matrix syntactic foam with a 10% volumetric filling fraction of Expancel 920 grade thermoplastic microspheres, adapted from^29^; **b** 3D rendering of the entire population of microspheres within the CT scan, showing the highly packed microstructure, adapted from^29^; **c** schematic of stress-strain curve of a glass microsphere syntactic foam under uniaxial compression showing three distinct stress-strain response regions; **d** stress-strain curve of a hollow thermoplastic microsphere (HTM) syntactic foam under uniaxial compression showing tunability of stress-strain response according to filler fraction, the inset shows the small strain response of the foam, adapted from ref. 40.

Syntactic foams are lightweight synthetic composites comprising a metal, ceramic, or polymer matrix and microsphere inclusions^1^. These materials are highly tailorable, as the matrix material along with the type, distribution, volume fraction and shell thickness of the microspheres can be independently adjusted to achieve bespoke, optimised mechanical properties^2–7^. The microstructure (see Fig. 1a, b, for example) plays a key role in the resulting macrostructural properties of the syntactic foam, including the mechanical response, which can be tuned to achieve a specific performance across a wide range of strain levels^7,8^. Syntactic foams are therefore employed across a broad range of applications including in the aerospace, automotive, marine, and sports equipment industries, and beyond^1,5,6,8–20^.

In general, high filling fraction syntactic foams exhibit complex load/unload behaviours. Under uniaxial loading, glass microsphere foams undergo three regimes of deformation during compression, as shown in Fig. 1c. Under small strain they respond in a linear elastic manner, with microspheres providing a stiffening mechanism. At medium strains, the macroscopic response exhibits a plateau region, due to the fracture of individual microspheres. At high strains, the material behaves linearly again due to densification of the foam, i.e., the filling of the microsphere cavities with debris. As such, the behaviour of these foams is non-recoverable, especially under extreme strains^21–23^.

Conversely, hollow plastic microspheres exhibit a similar stress-strain response but provide a recoverable large-strain response which is postulated to be due to the shells buckling under load, without fracture or failure. The nonlinear, large strain constitutive response can be highly tailored depending on the plastic microsphere type, volume fraction and shell thickness distribution^6,24,25^ (see example large and small strain responses in Fig. 1d). Despite buckling being postulated as the principal route for tailoring the nonlinear constitutive response, it has never been visualised in-situ. Validation of this mechanism is critical, because once understood it can be manipulated as a principal mechanism to tune the nonlinear response of the bulk material. Coupled with the microsphere volume fraction, size distribution and shell thickness distribution, this would give unprecedented control of the mechanical response of the foam.

Theoretical investigations into the buckling of a single embedded shell under small uniaxial compressive loading were conducted in a series of recent papers^26–28^. These models give an explicit formula for predicting both the critical stress at which buckling occurs and the buckling pattern, assuming linear elasticity in the pre-buckled state and perfect bonding between the shell and matrix. The buckling pattern was found to be governed by two key ratios: the ratio ζ = *h/R* of the shell wall thickness, *h*, and radius of the microsphere, *R*, and the ratio δ = µ_m_/µ_s_ of the shear moduli of the matrix, µ_m_, and shell, µ_s_^26,27^. These parameters are expected to be small in a syntactic foam, where the shells are typically thinner and stiffer than the matrix. When the value *η* = ζ/δ is not large, the expected buckling pattern under uniaxial compression is a band of axially symmetric ripples around the equator (where the ‘poles’ of the sphere are aligned with the direction of compression)^26,27^. This buckling pattern is expected to be observed despite possible shell imperfections or neighbour interactions, because the origin of the buckling emerges from a strongly heterogeneous pre-buckling stress in the shell. Conversely, if η is large (achieved by making the shell relatively thick or less compliant), the buckling should extend all over the shell, and the axial symmetry is expected to be lost. In this regime, the buckling patterns should typically have wavelengths comparable to the shell radius ^26,27^.

The differing responses of microspheres in different η regimes may significantly impact the bulk response of a syntactic foam. In particular, the persistent nature of equatorial buckling should cause most flexible microspheres to buckle in a geometrically coordinated manner. The morphologies of the microspheres at high strains are generally guided by the initial buckling pattern, leading to deformations being concentrated equatorially, which could result in a high degree of thermal, electrical and acoustic anisotropy in the bulk material. Conversely, full-shell (non-equatorial) buckling would not lead to this same degree of coordination, as the final deformed state will depend strongly on local imperfections and neighbour interactions. Therefore, we could, in principle, design bespoke syntactic foams with desired mechanical properties and loading curves by carefully selecting a mix of shell inclusions with specific critical buckling stresses, and which can either buckle equatorially or non-equatorially. Likewise, the spatial distribution of the microspheres within the foam could be tailored to suppress or enhance the coordination of any microsphere equatorial buckling under uniaxial loading.

Despite their direct impact on the mechanical response of syntactic foams, these predicted buckling regimes never been confirmed experimentally. Thermoplastic microspheres within syntactic foams have been imaged previously, but visualisation of uniaxial compression buckling patterns was not the aim of, nor achieved, in these studies^21,29–32^. Pre- and post-buckling states of hollow thermoplastic microspheres were recently visualised for syntactic foams under hydrostatic loading however (where different buckling modes are expected)^33^. Some evidence of buckling under uniaxial compression was provided at a larger length scale in ref. 34. where a polymeric shell (a table tennis ball) of 40 mm radius was embedded in an opaque polymer and then subjected to one uniaxial compression-decompression cycle. The shell was then excised from the polymer to observe the deformation pattern (which had remained due to the shell being deformed beyond its elastic regime). In at least one case, the shell had compressed equatorially as predicted by the theory, though in other cases more irregular deformations were observed. While these results suggest that equatorial buckling does indeed occur, in this case, there is a need to obtain more conclusive proof of the behaviour by observing the initial onset of this deformation in-situ. Theoretical results imply that the shell buckling pattern is a universal phenomenon that is independent of the shell size—the dependence is on the *dimensionless* ratios for shell thickness, ζ and shear moduli, δ.

To address this, and to confirm or disprove, the universality of the buckling of embedded shells, we conducted two in-situ tests of the equatorial buckling hypothesis, at macro and micro scales. The results confirm that equatorial buckling prevails whenever the microsphere shell wall is thin, relative to its diameter. Investigations at two different length scales indicate this effect is independent of the *absolute* size of the microspheres. Measurements of the buckling wavelengths confirmed the expected trends relative to ζ and δ, qualitatively. This suggests that the buckling wavelength under compression can be tuned by the appropriate selection of geometric and material parameters.

## Results and discussion

### Material parameters

The relevant dimensional and mechanical parameters for all of the tests, and the resulting ratios ζ and δ are given in Table 1. We also include the ratio η = ζ/δ, which can be used to predict the buckling pattern; with larger values corresponding to an increased likelihood of all-over (non-equatorial) buckling. Note that for the microscale experiments, the geometric values given are the mean values. In reality, the embedded spheres have a distribution of diameters and a distribution of shell thicknesses^29^. This results in a range of ζ, and therefore η, values across the population of microspheres within each sample (later we will compare microspheres with diameters within specific ranges).

**Table 1:** Mechanical moduli and shell dimensions of the tested material: List of the parameters which can be used to predict the buckling pattern of the embedded spheres under loading

Experiment	Matrix	Embedded Sphere(s)	Matrix Shear Modulus (µ_m_)	Shell Shear Modulus (µ_s_)	Mean Sphere Diameter (2R)	Mean Shell Thickness (h)
Macro	ClearFlex50 Polyurethane	Bralen RB	1.01 MPa	15.23 MPa	50 mm	0.760 mm
Micro	Silicone	Expancel 920	0.434 MPa	715 MPa	36.8 µm	0.291 µm
	ClearFlex50 Polyurethane	Expancel 920	1.01 MPa	715 MPa	36.8 µm	0.291 µm
	ClearFlex95 Polyurethane	Expancel 920	66.6 MPa	715 MPa	36.8 µm	0.291 µm
**Experiment**	**Matrix**	**Embedded Sphere(s)**	**Shell/Radius Dimensional Ratio (ζ =h/R)**	**Matrix/Shell Shear Modulus Ratio (δ = µ**_**m/**_**µ**_**s**_**)**	**η = ζ/δ**	
Macro	ClearFlex50 Polyurethane	Bralen RB	3.04 × 10^−2^	6.63 × 10^-2^	0.46	
Micro	Silicone	Expancel 920	1.58 × 10^−2^	6.07 × 10^−4^	2.61	
	ClearFlex50 Polyurethane	Expancel 920	1.58 ×10^−2^	1.41 ×10^−3^	1.12	
	ClearFlex95 Polyurethane	Expancel 920	1.58 × 10^−2^	9.31 ×10^−2^	0.17	

The measurement of these is discussed in the Methods section.

### Macroscale Experiment

**Fig. 2:**
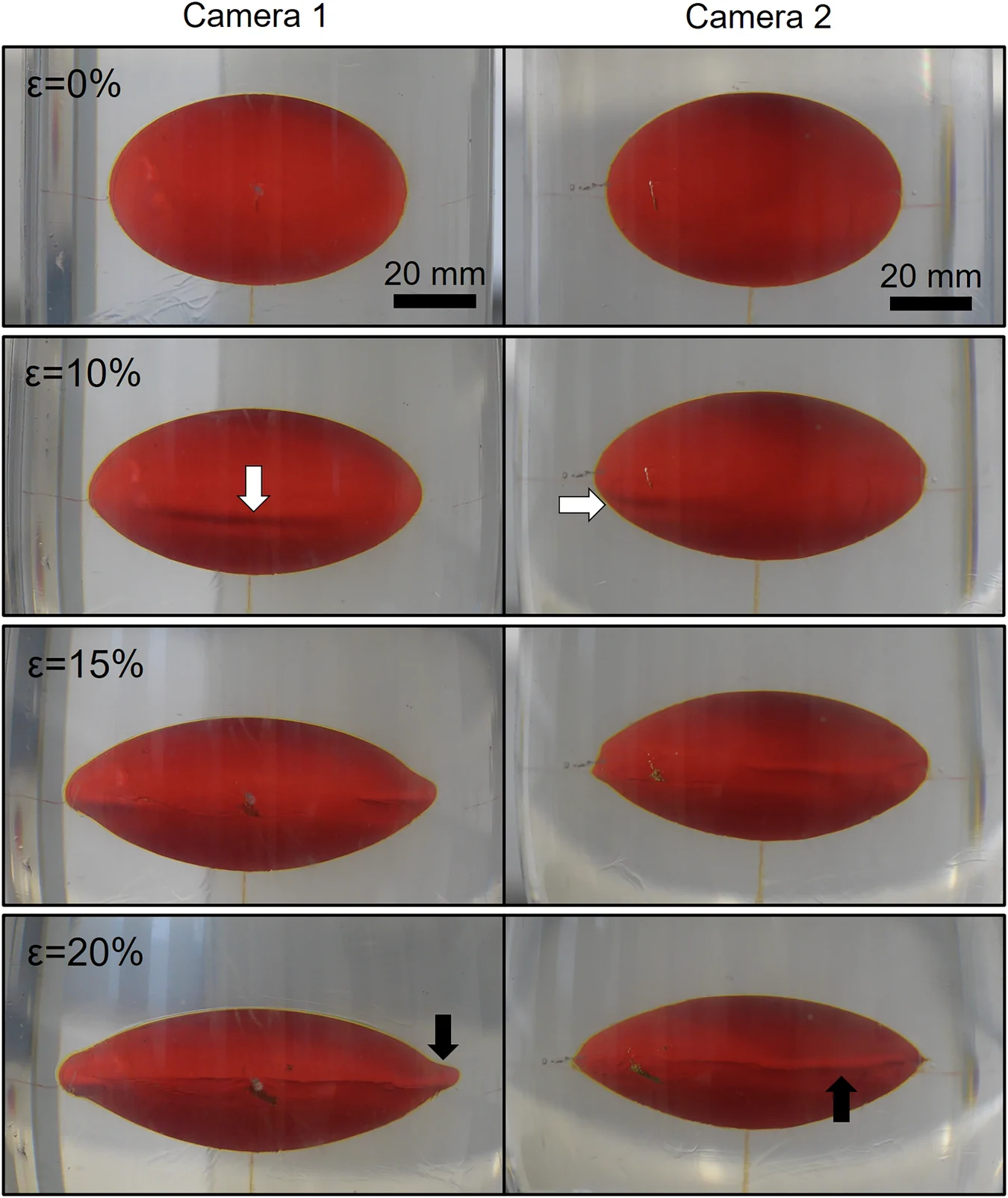
Buckling development in the macroscale experiment. Selected frames extracted from two video recordings of the deformation of an embedded polyurethane sphere. The perspectives of the two cameras were offset 135° from each other. The white arrow indicates equatorial buckling, which is clearly apparent at a compressive strain, ε, of 16%. This results in a pronounced cusp developing at higher strains (black arrows). As there is some optical lensing due to the cylindrical shape of the sample, the shape of the sphere is distorted, making it appear to be an ellipsoid even before deformation, and thus the scalebars should be taken as indicative only.

The macroscale experiment comprised the uniaxial compression of a 50 mm diameter polyethylene ball embedded within a transparent polyurethane matrix. Loading was applied slowly (0.05 mm/min; strain rate 6.94 × 10^−^^5^ s^−1^) up to a maximum compressive strain of 40%. Figure 2 shows frames extracted from two simultaneous video recordings of this test (included in the Supplementary Information). As indicated by the white arrows, at 10% strain, there is a clear rippling of the sphere concentrated around its equator, as predicted by theory for the specific values of ζ and δ in this case. The early frames of the videos confirm that this is not due to any (visible) pre-existing defect on the sphere surface and the continuous observation ensures the first (detectable) buckling has been observed. We do not observe any indication of matrix-shell debonding, even at high strains (~20%). This is the first such in-situ observation of embedded shell buckling at any scale, and was the precursor to the microscale experiment, at the more industrially-relevant length scale of the syntactic foam.

### Microscale experiment

The microscale experiment comprised in-situ x-ray computed tomography (CT) of syntactic foam samples under uniaxial compression. Expancel 920 microspheres were embedded within three different matrices (Silicone, ClearFlex50 and ClearFlex95) at a low (nominally 0.5%) volumetric filling fraction. Full details of these materials are given in the Methods section. Compressive loading was incremental in small steps from 0% strain to a level considerably beyond the onset of buckling (up to 20–35%) using a loading speed of 0.02 mm/min (equivalent to a strain rate of 2.78 × 10^−^^5^ s^−1^). A full tomogram (pixel size 0.65 µm) was collected at numerous *static* strains, during both loading and unloading of each sample (see Methods section), allowing the first onset of buckling to be imaged.

**Fig. 3:**
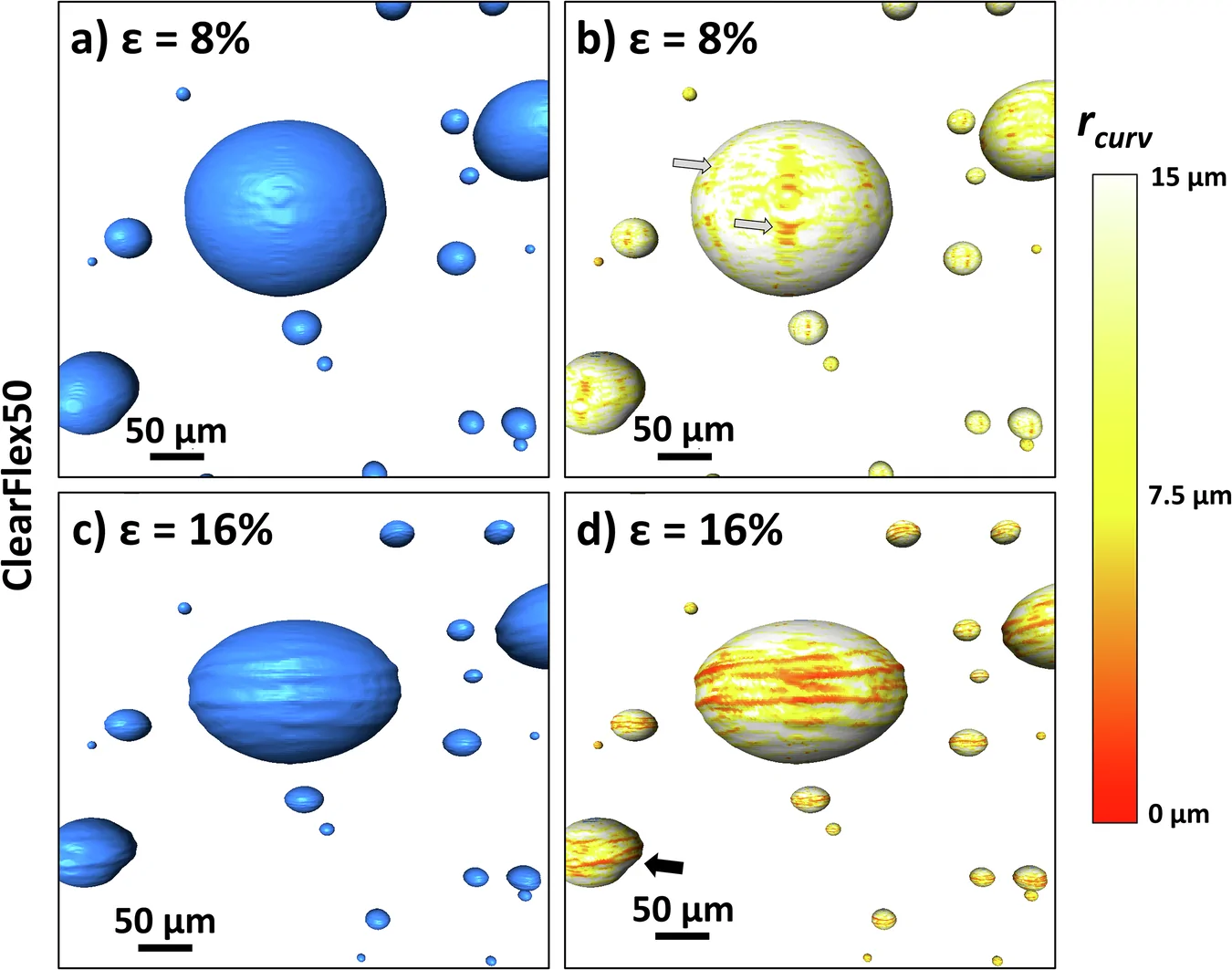
Microsphere buckling during uniaxial loading. Visualisation of Expancel 920 microspheres in ClearFlex50 under uniaxial strain, before and after crossing the critical buckling threshold: **a**, **c** surface renderings of the microspheres, showing rippling along the equator at the higher strains, **b**, **d** surface renderings with radius-of-curvature (*r*_curv_) colouring to further accentuate the equatorial buckling. The black arrow indicates an off-equator defect present on one of the microspheres. The vertical fringes (highlighted by the grey arrows) are artefacts of the surface generation.

Figure 3a, b shows surface renderings of a selected microsphere in the ClearFlex50 matrix, after segmentation from the reconstructed X-ray CT data, at two different strain levels. Here, and in all subsequent figures, the compression axis is vertical and ε denotes the bulk compressive strain of the sample. As in the macroscale experiment, a clear rippling of the microsphere shell is seen around the equatorial region at elevated strain. Curvature colour mappings are shown in Fig. 3c, d, which indicate the radius (*r*_curv_) of a sphere which locally fits the surface renderings (i.e., the reciprocal of the maximum curvature, in any direction). These colour mappings are provided to highlight areas where the microsphere deviates from an ellipsoidal shape, i.e., where the shell has buckled.

The images at low strain (ε = 8%) suggest that the buckling observed at higher strain is likely the first buckling mode to emerge for these microspheres under uniaxial compression. The possibility of an earlier non-equatorial buckling mode below our resolution limit cannot be excluded, but this is not theoretically predicted^26,27^ in this case, nor observed in the macroscale experiment. The images at low strain (ε = 8%) also show that the equatorial buckling observed is not due to a (detectable) pre-existing defect, as none are present for the central microsphere in these images. Furthermore, as highlighted by the black arrow in Fig. 3d, the buckling pattern emerges in a nearby microsphere despite an off-equator defect, showing that the equatorial buckling is a robust effect. The vertical fringes visible in Fig. 3b (highlighted by the grey arrows) are artefacts of the surface generation process (described in the Methods section), rather than a form of buckling. This is clear when looking at ε = 0% images, where these fringes are still present; see Figs. S3–5 in the Supplementary Information.

No evidence of matrix-shell debonding was detected in any of the three matrices (up to ε = 20%). The microsphere shells are thin and similar in density to the matrix material and therefore cannot be resolved directly from these X-ray CT images; whereas the microsphere interior (air) provides good contrast. The detection of the buckling pattern implies the matrix-shell interface remains relatively intact (otherwise the microsphere would act as a cavity, and the X-ray CT images would reveal a regular compressed ellipsoid shape). The possibility of partial debonding cannot be excluded however, which may explain the slight mismatch between the theoretical and measured buckling wavelengths (discussed later).

Similar figures for selected microspheres in the ClearFlex95 and Silicone matrices are available in the Supplementary Information (Figs. S1 and S2). The strain levels shown (ε = 8% and 16%) were chosen as they reveal the unbuckled and buckled states for the majority of the larger microspheres in all three matrices, allowing a comparison between matrices at the same absolute strains. Rather than the initial state, the unbuckled state at ε = 8% compressive is shown to highlight that the microspheres do not buckle immediately under strain – there is a critical buckling strain for each microsphere which is related to ζ and δ. Radius-of-curvature colour mappings for the entire population of microspheres in each matrix, at a range of strains (including ε = 0%), are provided in the Supplementary Information in Figs. S3–S5. These show that the equatorial buckling pattern is repeated across the entire population of microspheres and persists at higher strain levels. For smaller microspheres (≲20 µm diameter), buckling is not observed at any strain level, likely due to the image resolution limit.

To further test the agreement of our experiments with the theory, we explore two key predictions from the theory. These are (i) that within a given matrix, larger diameter spheres (smaller ζ) should have a smaller buckling wavelength (relative to the sphere diameter) and (ii) for microspheres of a given size, those embedded in a stiffer matrix should have a smaller buckling wavelength. Note: for trend (i) we assume a fixed shell wall thickness for all microspheres, such that the trend can be assessed in terms of the microsphere diameter only, rather than the parameter ζ = *h/R*. Previous measurements of Expancel 920 microspheres revealed an average shell wall thickness of ~290 nm, with no dependence on the microsphere diameter ^29^.

**Fig. 4:**
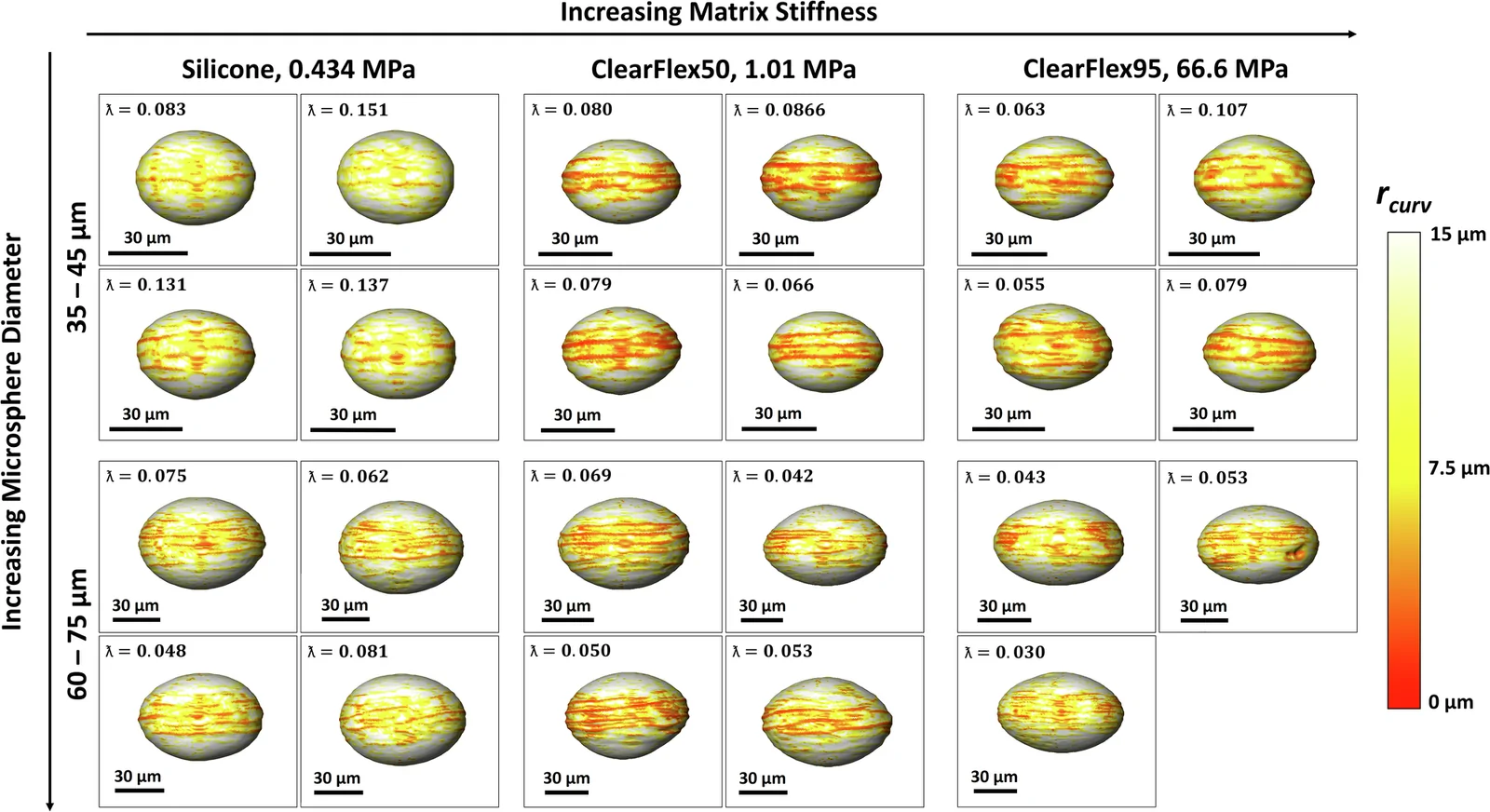
Effect of microsphere diameter and matrix stiffness on buckling wavelength. Surface renderings of representative microspheres, in each matrix, with diameters within specific ranges. A radius-of-curvature colouring is used for the renderings to highlight buckling. Each microsphere is shown at compressive strain of 16%. The measured normalised wavelength ƛ is shown on each figure.

**Fig. 5:**
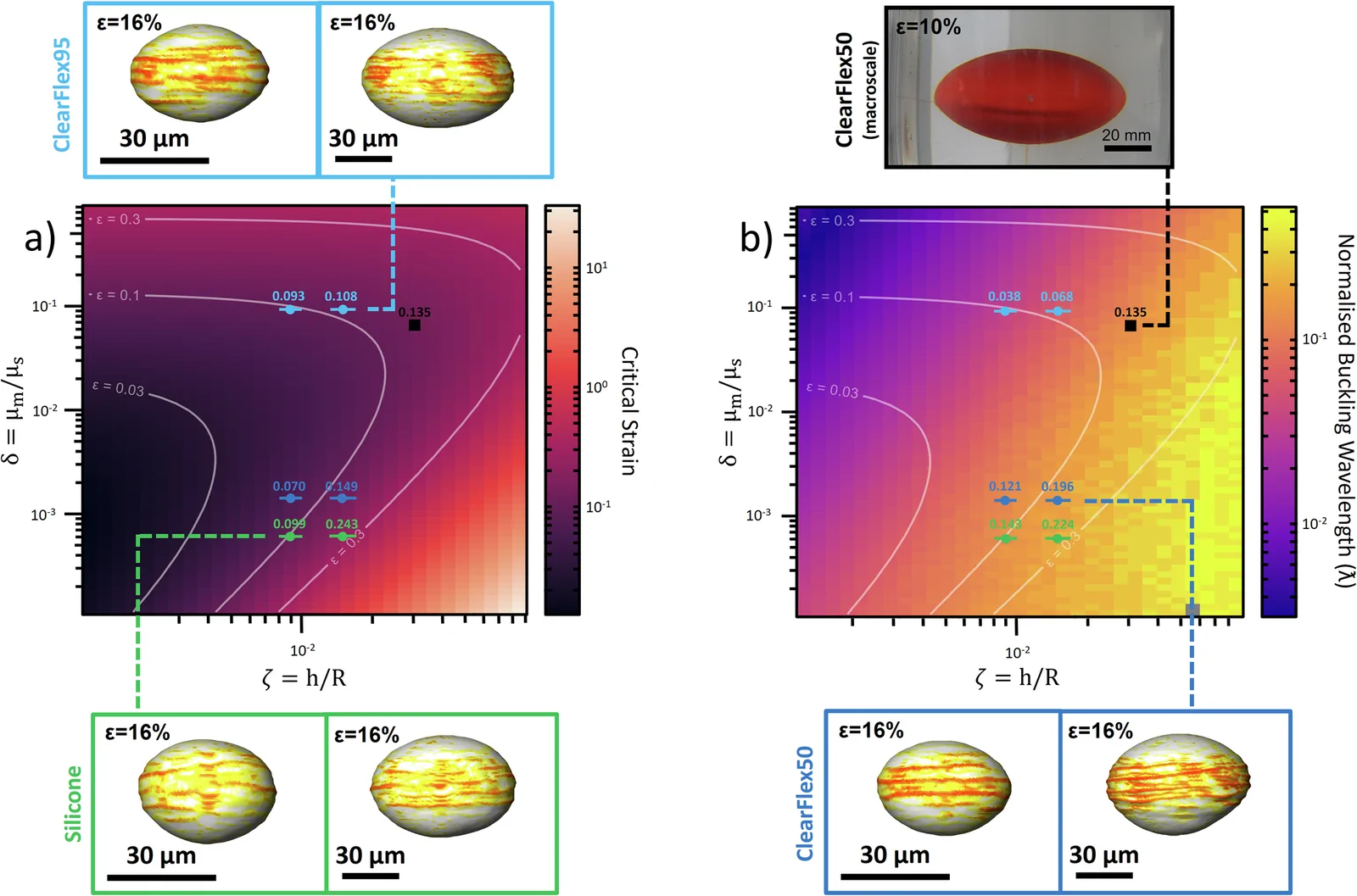
Comparison of experimental results to theory. Heatmap plots showing predictions of the critical buckling strain (**a**) and normalised buckling wavelength (**b**) under uniaxial compression, for a range of different stiffness, ζ, and thickness, δ, ratios. The three microscale experiments are plotted as white dots with the bars indicating the spread in ζ for the two comparative diameter ranges used (35 − 45 µm and 60–75 µm) The square marker indicates the macroscale experiment. The numbers accompanying the markers indicate the predicted value of buckling wavelength or critical strain, according to the methods presented in ref. 26. For each matrix/sphere combination an example image showing buckling is provided.

The trends described above are explored in Fig. 4 which shows representative microspheres within two diameter ranges (35–45 µm and 60–75 µm), when held at ε = 16%, for each matrix. These size ranges were selected to ensure a few microspheres in each matrix could be shown (in ClearFlex50, only three microspheres were within the larger diameter range) whilst avoiding the smallest microspheres whose buckling patterns cannot be discerned reliably due to the finite imaging resolution. As the trends described above are relative to the sphere diameter, the microspheres in Fig. 4 are scaled to the same size, to make comparison easier. For each microsphere, the buckling wavelength λ was calculated from as many peaks as could be easily discerned in each image (generally 5–9) and then normalised against the microsphere diameter (using the mean of the horizontal and vertical axes). These values are given in Fig. 4 for each microsphere and as averages in Table 2, and show that, *qualitatively*, our results agree with the trends described above. The normalised wavelength, ƛ, according to predictions from theory, as described below, are also given in Table 2. Values of *r*_curv_ at different strain levels or for different microsphere sizes are not provided here as the colour mappings (Figs. 3–5, S1–5) are provided simply to better highlight the buckling pattern.

**Table 2:** Mean normalised buckling wavelength measurements: Measured and predicted values of ƛ, for a selection of microspheres, within the two selected diameter ranges, embedded within three different matrices and compressed to a strain of 16%

Matrix	Microsphere Diameter (D) Range	Measured Normalised Wavelength (*ƛ*=λ/D)	Predicted Normalised Wavelength (*ƛ*=λ/D)
Silicone (0.434 MPa)	35–45 µm	0.100 ± 0.015	0.224
	60–75 µm	0.058 ± 0.007	0.143
ClearFlex50 (1.01 MPa)	35–45 µm	0.075 ± 0.010	0.196
	60–75 µm	0.049 ± 0.005	0.121
ClearFlex95 (66.6 MPa)	35–45 µm	0.063 ± 0.008	0.068
	60–75 µm	0.035 ± 0.003	0.038

The measured ƛ values were computed from the microspheres shown in Fig. 4.

Figure 5 shows the dependence of buckling wavelength, *ƛ,* (a) and critical buckling strain, ε_crit_, (b) on the dimensionless parameters ζ and δ, according to the theoretical models in ref. 26. as a heatmap. The colour on each plot indicates the predicted values of wavelength and buckling strain and the grey contours indicate specific values of critical buckling strain. For each of the experiments described here, the corresponding point in ζ and δ is marked and an example image is provided. In agreement with the theoretical model, an equatorial buckling pattern is evident in each case. The numbers displayed denote the predicted values for *ƛ* or ε_crit_.

The trends in buckling wavelength predicted theoretically (i.e., a smaller wavelength for shells with a larger diameter or embedded within stiffer matrices) are observed in our experiments but there are some quantitative differences between the predicted and measured values. With reference to Table 2, we find a good agreement for ClearFlex95 but only see a qualitative agreement for the other matrices. At present it is not known why there is a discrepancy in the measured and theoretical values of *ƛ* for the other two matrices but, as noted previously, the trends with respect to ζ and δ appear valid. It is not possible to quantify a value for the critical buckling strain from the experimental data, due to the resolution of the imaging techniques. To observe buckling, the microspheres must first be strained by an unknown quantity *beyond* the onset of buckling until the amplitude of buckling can be resolved. This is not an issue for the buckling wavelength measurement however, as only the amplitude is expected to increase with strain increases beyond the onset of buckling.

## Conclusion

The work presented here has experimentally confirmed the multiscale, equatorial buckling of thin, stiff embedded microspheres - a result that has previously only been predicted theoretically. This was achieved in three different matrices with dilute distributions of microspheres, at relatively low strain rates of ~10^−5^ s^−1^ (see ref. ^35^). For more densely packed syntactic foams, further work is required to understand the effects of near-neighbour interactions on buckling. At higher strain rates that may be encountered in service, viscoelasticity may also play an important role in the buckling response^35^. Nonlinear viscoelastic models for syntactic foams and closely related complex materials remain at a relatively early stage of development however^35^. The results presented here are an important step in understanding the nature of microsphere buckling in syntactic foams and related composites and metamaterials and may have profound implications for materials design. Indeed, they open new avenues for the exploration of soft materials with controlled nonlinearity. Beyond mechanical applications, the ability to precisely control the buckling response (via the parameters ζ and δ and/or the applied load) opens up the intriguing possibility of thermoplastic syntactic foams as responsive photonic materials^36,37^. Such mechanochromic materials can significantly change their optical properties in response to strain, with possible applications in strain sensing^38^.

## Methods

### Testing of matrix materials

Small cylindrical samples of the three matrix materials were prepared, according to the formulation specifications described below, to experimentally determine material properties, given in Table 3. No microspheres were embedded within these samples. Testing was done using an Instron 5569 mechanical testing machine using a displacement rate of 10 mm/min with the stress/strain values recorded at a rate of 10 Hz. Due to noise in the data, from the testing equipment, at low strains, a Gent model was fitted to this data to extract the infinitesimal shear modulus. For each matrix, three samples were tested and the median value of the three shear moduli was used as the representative value.

**Table 3:** Mechanical properties of the three matrix materials used as the microsphere host media: These values were determined by mechanical testing, as described above

Cylinder material	Sample dimensions		Shear Modulus
	Diameter (mm)	Height (mm)	(MPa)
ClearFlex50	11.90	14.60	1.01
ClearFlex95	13.70	11.95	66.6
Silicone	13.70	11.95	0.434

### Macroscale experiment

A cylindrical sample (height = diameter = 120 mm) was fabricated by embedding a single hollow sphere (Bralen RB 03-23 low density polyethylene, diameter 50 mm, thickness 0.76 mm; Precision Plastic Ball Company) within an elastomeric matrix (ClearFlex50 polyurethane; Bentley Advanced Materials) as it cured within a polycarbonate mould. This matrix was selected due to its high transparency, allowing the deformation of the embedded sphere to be observed in-situ, as described below. Prior to pouring the matrix into the mould, it was thoroughly degassed in a vacuum chamber. Four metallic pins were placed through the mould wall to keep the embedded ball central and were removed once the viscosity increased to a sufficient level for the ball to remain in place, but low enough for the matrix material to refill the gaps after the pins were removed. The curing was otherwise according to the manufacturer specifications with the sample removed from the mould after 24 h. The shear moduli of the matrix and embedded sphere material were 1.01 and 15.23 MPa, respectively. The Poisson’s ratio of the shell material was estimated from ref. 39. as ${v}_{s}=0.43$. The matrix is assumed to be incompressible^24^ with ${v}_{m}=0.49$ used based on results in ref. ^40^.

**Fig. 6:**
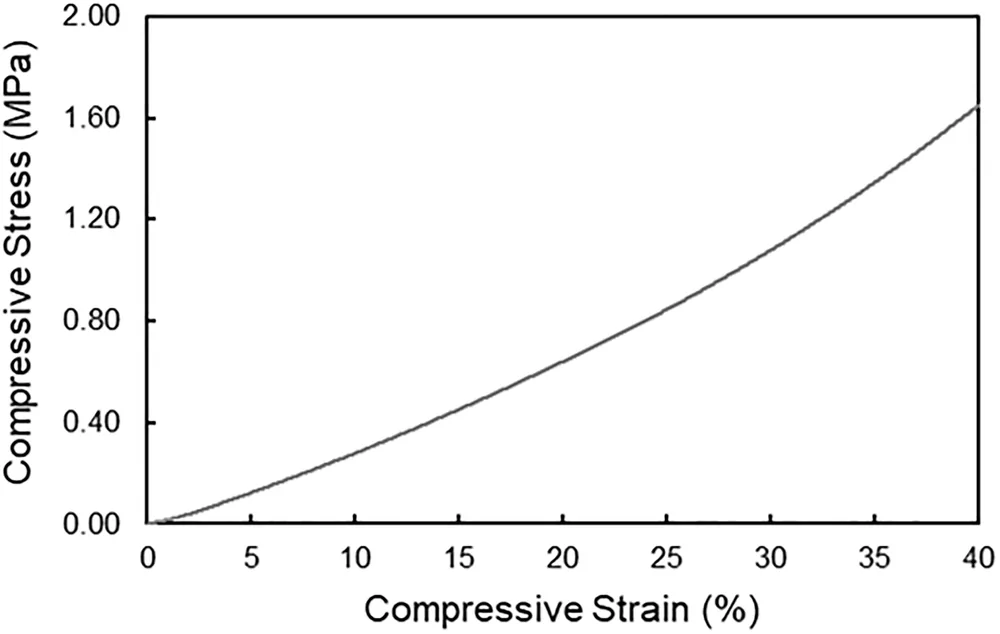
Mechanical response of the macroscale experiment sample. Mechanical test data acquired for the macroexperiment sample showing the nominal stress vs strain during the video-recorded compression test, as recorded by the Instron 5569 testing machine.

Uniaxial compressive strain was applied to the sample using an Instron 5569 mechanical testing machine; with the sample imaged in-situ, as described below. Samples were strained to a peak strain of 40% at a continuous strain rate of 5 mm/min. The corresponding load/displacement data was recorded at a frequency of 50 Hz and is plotted in Fig. 6

During the loading described above, the samples were recorded using two Panasonic Lumix GH5 cameras at 25 fps (see video file in Supplementary Information). The cameras were positioned to record at approximately the same height on the sample but were offset 135° from each other to provide complementary overlapping perspectives. Selected frames, labelled with the corresponding strain levels are shown in Fig. 2.

### Microscale experiment

Cylindrical samples (diameter = 14 mm, height = 12 mm) comprising different polymeric matrices with embedded hollow thermoplastic microspheres were fabricated according to the procedures described in ref. 29. The specific microspheres used were ‘Expancel 920 DE 80 d30’ from Nouryon. Three different matrices were used to provide a range of shell/matrix stiffness ratios (see Table 1) such that the resulting effect on buckling behaviour, if any, could be observed. The Poisson’s ratio of the shell material was inferred from modelling results in ref. 29. as ${v}_{s}=0.2375$. The matrices are assumed to be incompressible^24^ with ${v}_{m}=0.49$ used based on results in ref. ^40^.

The intent was to achieve a low volumetric microsphere filling fraction of approximately 0.5% for each sample. However, due to the difficulties in weighing such a small quantity of microspheres, the real filling fractions (according to analysis of the X-ray CT images) vary from 0.19 to 1.12%. Regardless, a relatively large average distance between the microspheres was still achieved (the shortest average, for Silicone, being 194 µm which is ~4.8 mean shell radii), allowing near neighbour effects to be safely ignored.

**Fig. 7:**
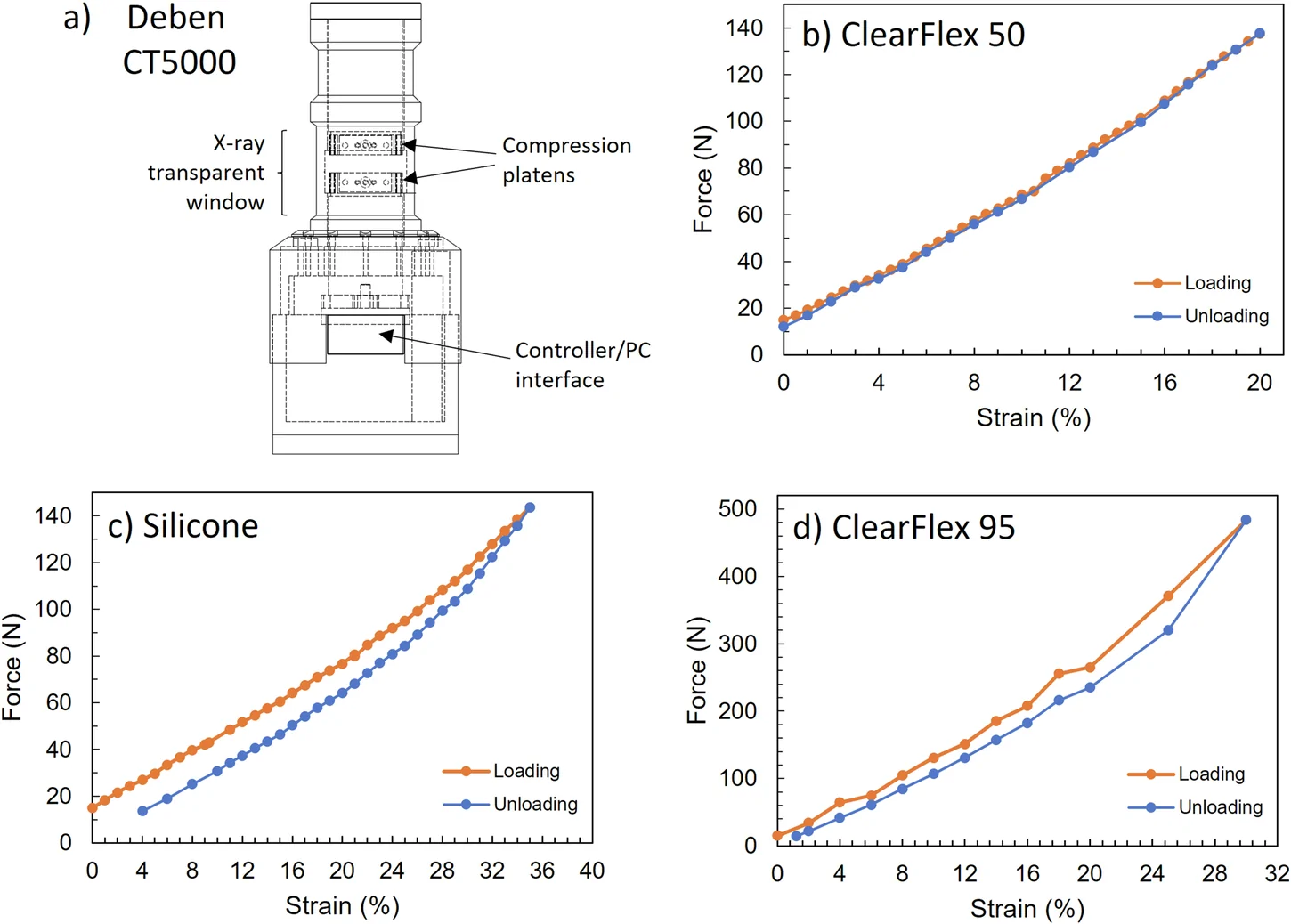
Summary of mechanical loading of microscale experiments. **a** depiction of the Deben CT5000 used to apply uniaxial compression; **b**–**d** Stress-strain curves, as recorded by the Deben CT5000, for the three matrices filled with a 0.5% volume fraction of Expancel 920 microspheres. Compression was held for ~ 10 minutes at each of the points marked, to allow time for sample relaxation and a full X-ray CT scan.

Compressive strain was applied to the microsphere samples using a Deben CT5000 test rig (depicted in Fig. 7a) fitted with a 5 kN loadcell. This rig is designed for use in in-situ CT experiments and has an X-ray-transparent window through which the sample can be imaged. For each sample, imaging was conducted at a range of *static* compressive strains. Generally, the method comprised fine strain steps (~0.5–1%) during loading and coarser steps (~1–2%) during unloading. This was to ensure imaging was done as close to the first emergence of microsphere buckling as possible; noting that we only have theoretical estimates of the critical buckling strain. After buckling was observed, the coarser unloading strain steps allowed the beamtime to be more efficiently utilised, whilst still obtaining some information about the recovery of the microspheres. The loading and unloading steps for each sample are given in Fig. 7b–d. In all cases, the strain rate for loading and unloading was 0.02 mm/min. Prior to imaging, the samples were allowed to settle at the target strain level for at least ten minutes or until the force reading from the test rig was stable–whichever was longest. This was done to avoid any blurring associated with sample relaxation during scanning. Scans were reconstructed and checked for blurring prior to continuing the test schedule, with repeat scans performed when necessary.

**Fig. 8:**
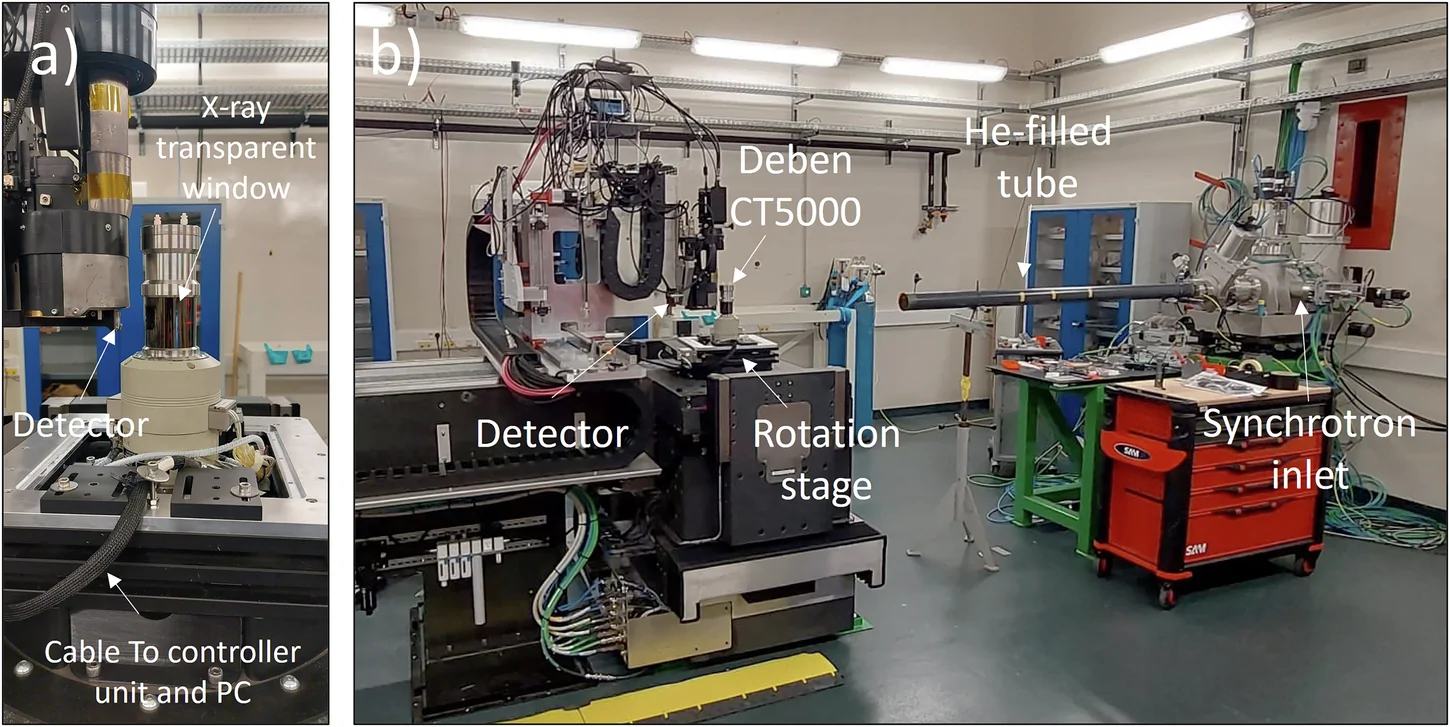
Photographs of the microscale experiment set-up. **a** close-up showing the detector and the Deben CT5000 mounted on the beamline rotation stage, **b** the ANATOMIX beamline during our experiments.

X-ray CT imaging was conducted at the ANATOMIX beamline of Synchrotron SOLEIL in Saint-Aubin, France, in the second experimental hutch of the beamline^41^. For all samples, a polychromatic (“white”) X-ray beam with a central photon energy around 35 keV was used. The beam was filtered (100 µm Cu, 26 µm Au) before the sample and rig to achieve a final transmission of ~20%. A Helium-filled tube was used to reduce beam attenuation across the ~5 m air path between the vacuum exit window and the rotation stage, onto which the rig containing the sample was attached. The experimental set up is shown in Fig. 8. For every scan, 2000 radiographs, each with an exposure time of 150 ms were collected, covering 180° of sample rotation. For this, a 2048 × 2048-pixel CMOS-based detector (Hamamatsu Orca Flash 4.0 V3) with a physical pixel size of 6.5 µm was used. The detector was coupled to a single-crystal scintillator screen (lutetium aluminium garnet, supplier: Crytur, Turnov, Czech Republic) via 10x magnification optics (Mitutoyo microscope objective M PLAN APO 10x/NA 0.28), giving a pixel size of 0.65 µm and a ~1.3 × 1.3 mm field of view for the projections.

**Fig. 9:**
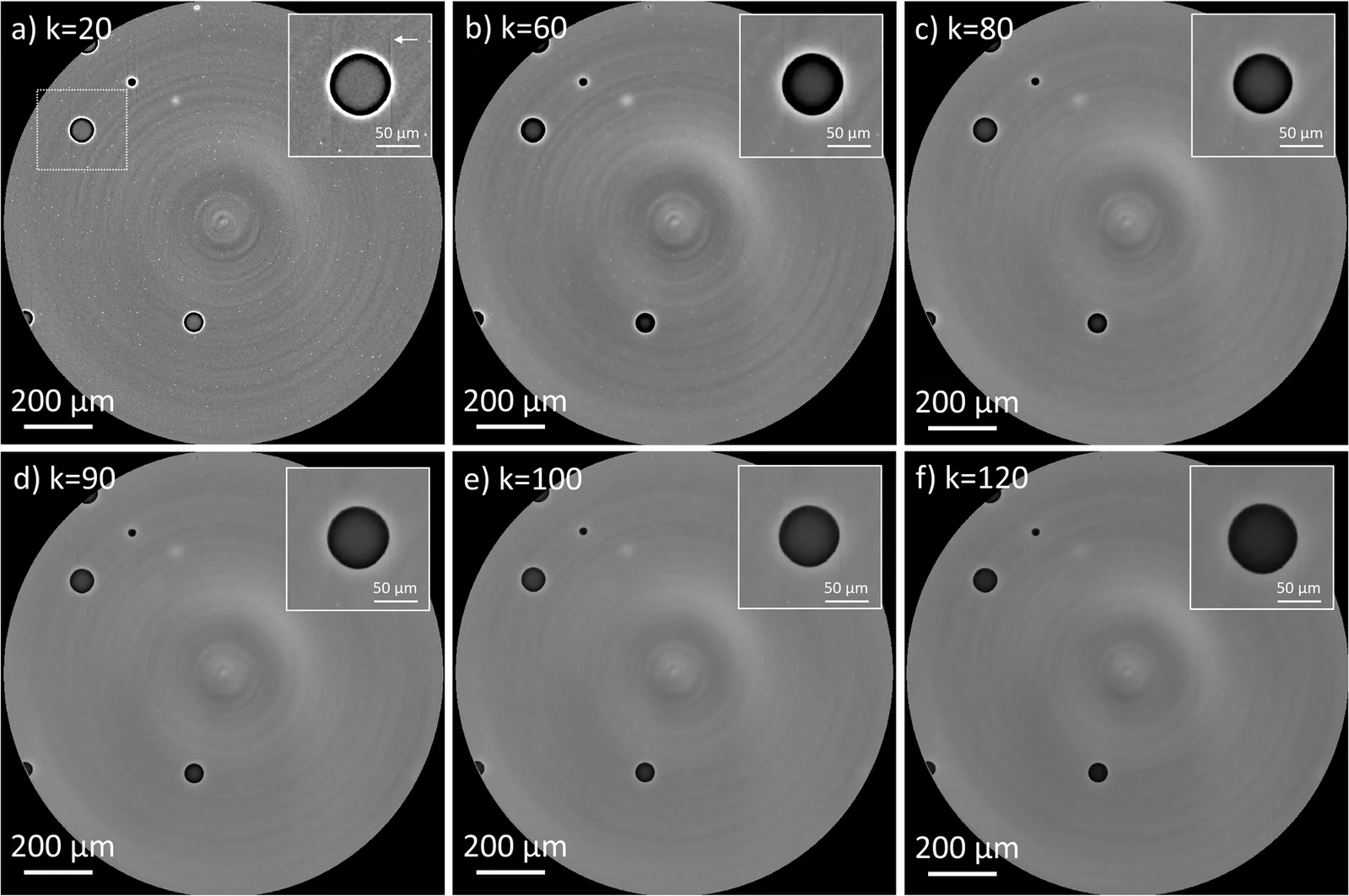
Depiction of the optimisation process for the Paganin filter kernel size, k. **a**–**f** show, for the ClearFlex50 sample, the central slice of each of the CT reconstructions at different values of k. The insets show the sphere highlighted in (**a**) at higher magnification. The value of *k* was chosen to minimise the phase fringes (see white arrow in the inset of (**a**)), around each microsphere whilst preserving image clarity.

The projections were reconstructed via filtered backprojection, using the standard data processing pipeline at the beamline, based on in-house software for preprocessing^42^ and using PyHST2 software (ESRF, Grenoble, France)^43^ as the backend for tomographic reconstruction. A Paganin filter^44^ was applied during reconstruction to improve the phase-contrast (i.e., the shell-matrix contrast). The Paganin kernel size, *k*, was optimised and fixed for all scans for each sample (Silicone: 100 pixels, ClearFlex50: 80 pixels, ClearFlex95: 100 pixels). Figure 9 depicts this optimisation for the Silicone matrix, as an example, which shows that the image quality is relatively insensitive to changes in *k* close to the values which were ultimately selected. We estimate that the true resolution in our reconstructed scans is approximately 1.95 µm (i.e., using three times the pixel size as a rule of thumb).

All of the tomographic datasets were processed and visualised using Avizo2025.1 software. Segmentation of the microspheres was achieved via the procedure described in ref. 29. The visualisations (i.e., Figs. 3, S1, 2) of the segmented microspheres were achieved via the ‘Generate Surface’ and ‘Surface Rendering’ modules in Avizo. The radius-of-curvature colour mappings (Figs. 3–5, S1–S5) were generated by using the ‘Curvature’ module, in Avizo. Microspheres belonging to two different diameter ranges were selected using the ‘Filter by Measure’ module in Avizo using the ‘EqDiameter’ measure (the diameter of a sphere with the same volume as the segmented object–which here are the compressed ellipsoidal microspheres).

## Supplementary information

Transparent Peer Review file

Supplementary Results

## Data Availability

The X-ray CT data (Figs. 3–5), macroscale experiment video (Fig. 2) and predicted buckling parameters (Fig. 5) that support the findings in this study are available in a figshare repository with the identifier [10.48420/32133037].

## Code availability

The codes used to generate the parameter values presented in Fig. 5 are available in a figshare repository with the identifier [10.48420/32133037].
