# Supplementary material for: Buckling of elastomer-embedded thermoplastic microspheres under uniaxial compression revealed by in-situ X-ray micro-CT
Source: Commun Mater. 2026 Jun 5;7(1):236. doi: 10.1038/s43246-026-01214-w (PMC13561867; doi:10.1038/s43246-026-01214-w)
Supplement: Supplementary file 1 [file 43246_2026_1214_MOESM1_ESM.pdf]

nature portfolio

Peer Review File

Buckling of elastomer-embedded thermoplastic microspheres under uniaxial compression revealed by in-situ X-ray micro-CT

Corresponding Author: Dr Matthew Curd

This file contains all editorial decision letters in order by version, followed by all author rebuttals in order by version.

Version 0:

Decision Letter:

** Please ensure you delete the link to your author homepage in this email if you wish to forward it to your coauthors **

Dear Dr Curd,

Thank you for submitting your manuscript, “Buckling of embedded microspheres under compression revealed by in-situ X-ray micro-CT", to Communications Materials. It has now been seen by 3 referees, whose comments are appended below. You will see that while they find your work of potential interest, they raise substantial concerns that must be addressed. In light of these comments, we cannot accept the manuscript for publication but are interested in considering a revised version that addresses these serious concerns.

We hope you will find the referees' comments useful as you decide how to proceed. Should additional data or analysis allow you to address these criticisms, we would be happy to look at a revised manuscript.

To allow us to move forward with your work, we also ask that you edit your manuscript according to the table link provided below. **Please read this document carefully as we will be unable to further assess your revised paper until these important points are addressed.**

Please outline all revisions made in the right-hand column.

Table link:

https://springernature-my.sharepoint.com/:w:/r/personal/vau0656_springernature_com/Documents/COMMSMAT-25-1041-T Matthew Curd.docx?d=wd39e7bb90afc41c59a14d29efa7672d6&csf=1&web=1&e=P4FE9w

When resubmitting, please also include:

- A point-by-point response to the reviewers’ comments. If you are unable to address specific reviewer requests or find any points invalid, please explain
- A clean version of your revised manuscript with no mark-ups
- A marked-up version of your paper with all changes highlighted in a different colour

Please use the link below to submit your revised files:
Link Redacted

** This url links to your confidential home page and associated information about manuscripts you may have submitted or that you are reviewing for us. If you wish to forward this email to co-authors, please delete the link to your homepage first **

We hope to receive your revised paper within twelve weeks, but we understand that revisions may take longer. Please let us know if you find that the revision process will take substantially more time.

We are committed to providing a fair and constructive peer-review process. Please do not hesitate to contact me if you have any questions or would like to discuss these revisions further. We look forward to seeing the revised manuscript and thank you for the opportunity to review your work.

Best regards,

John Plummer, PhD
Chief Editor
orcid.org/0000-0003-4824-8497
Communications Materials

Reviewers' comments:

Reviewer #1: see attached file.

Reviewer #2 (Remarks to the Author):

I co-reviewed this manuscript with one of the reviewers who provided the listed reports. This is part of the Communications Materials initiative to facilitate training in peer review and to provide appropriate recognition for Early Career Researchers who co-review manuscripts.

Reviewer #3 (Remarks to the Author):

This paper focuses on the compressive buckling behavior of embedded microspheres in syntactic foams, verifying the theoretically predicted equatorial buckling mode through multi-scale in-situ experiments. It clarifies the correlation between buckling behavior and dimensionless parameters ζ (shell thickness/radius) and δ (matrix/shell shear modulus ratio), providing critical experimental support for regulating the mechanical properties of composite materials. The research topic holds theoretical value and application prospects. However, before recommending this manuscript for publication, the authors should address the following points to strengthen its rigor and clarity.
1. In Table 2, the measured normalized buckling wavelengths of shells in Silicone and ClearFlex50 matrices are notably lower than the theoretical predictions, with good agreement only observed for the ClearFlex95 matrix. The paper attributes this discrepancy to “the linear elasticity assumption being more valid in rigid matrices,” which lacks adequate rigor. As illustrated by the stress-strain curve of the ClearFlex50-matrix composite (Figure M2(b)), the material exhibits excellent elastic linearity within the tested strain range. The authors should discuss additional potential factors to strengthen the rationality of the explanation.
2. The paper does not clarify the interface state between the shells and the matrix during buckling (e.g., perfect bonding, partial debonding, or slip). It remains unclear whether interface strength or bonding quality affects the initiation of buckling, the evolution of buckling mode, or the critical buckling strain. Supplementary characterizations of the shell-matrix interface are recommended to clarify the role of interface in buckling behavior.
3. According to the theoretical framework, the buckling modes (equatorial buckling and all-over buckling) are governed by the dimensionless parameters ζ and δ. In Figure 5, some experimental data points lie close to the theoretical boundary between the two buckling modes, yet the shells consistently exhibit typical equatorial buckling characteristics. This raises questions about the accuracy of the theoretical boundary or the influence of unaccounted factors in the experiments.
4. The authors emphasize that the nonlinear response of syntactic foams can be modulated by tuning the dimensionless parameters ζ and δ, and further propose potential applications in soft robotics and strain sensing. However, the manuscript lacks quantitative design schemes that bridge theoretical parameters, material properties, and target application performance, leaving a gap between basic research findings and practical engineering applications.
5. Figure 4 scales microspheres of different sizes to the same dimensions for comparative convenience, but this conceals the intuitive impact of actual size differences on buckling wavelength. The authors are advised to clearly indicate the scaling ratio in the legend to avoid misinterpretation by the readers.
6. This paper qualitatively depicts buckling regions via curvature color mapping but lacks quantitative curvature data. The authors are suggested to plot curves of curvature changes with strain to quantify the evolution of buckling degree.
7. A recent study on syntactic foam failure behaviors (European Journal of Mechanics-A/Solids, 2024, 105: 105256) aligns closely with the research theme and is recommended for appropriate citation.
8. This paper assumes all matrices have a Poisson’s ratio of 0.49 (based on the incompressible assumption), while no experimental basis or justification is provided for this assumption.

** See Nature Research's author and referees' website at www.nature.com/authors for information about policies, services and author benefits

Version 1:

Decision Letter:

** Please ensure you delete the link to your author homepage in this email if you wish to forward it to your coauthors **

Dear Dr Curd,

Thank you once again for submitting your manuscript, “Buckling of elastomer-embedded thermoplastic microspheres under uniaxial compression revealed by in-situ X-ray micro-CT," to Communications Materials. Your manuscript has been seen again by the referees, whose comments are appended below. Reviewer 2 confirmed in comments to the editors that their requests had been addressed, and we only require some minor amendments before we can accept your paper.

Our remaining requests are:
-Please consider Reviewer 3's comment regarding the use of "buckling" in the title.
-Please add a 'Conclusion' heading at line 312.

-Please provide supplementary information file in PDF format.
-Please ensure that captions for Supplementary display items reference all panels.

This will be the final revision of your manuscript. We ask that you carefully review all files associated with your paper and follow the link below to upload the final version of all files, including display items and supplementary material. Please ensure that these files are clean, without any markups or comments, as they will be sent for publication.

Link Redacted

We hope to receive this updated version of your paper within 1 week, but please let us know if you find that you need more time.

Best regards,

John Plummer, PhD
Chief Editor
orcid.org/0000-0003-4824-8497
Communications Materials

Reviewers' comments:
Reviewer #2 (Remarks to the Author):

I co-reviewed this manuscript with one of the reviewers who provided the listed reports. This is part of the Communications Materials initiative to facilitate training in peer review and to provide appropriate recognition for Early Career Researchers who co-review manuscripts.

Reviewer #3 (Remarks to the Author):

The authors addressed my previous comments well. I recommend its publication with one final minor remark.

Since the authors adopted the term of 'Buckling ...' in the title, do we suppose to see a fully recoverable configuration for those spherical shells? If not, words like 'collapse', 'damage', 'failure' could be more precise.

Version 2:

Decision Letter:

Dear Dr Curd,

We are delighted to accept your manuscript titled "Buckling of elastomer-embedded thermoplastic microspheres under uniaxial compression revealed by in-situ X-ray micro-CT" for publication in Communications Materials. Thank you for

choosing to publish your interesting work with us.

Acceptance of your manuscript is conditional on all authors' agreement with our publication policies. In particular, your manuscript must not be published elsewhere and there must be no announcement of the work in the media until the publication date.

Please carefully read the information below for information on what to expect from the next steps of the publishing process:

Article in Press:

Please note that in advance of your paper being published we will host an early access version, known as an ‘Article in Press,’ on our journal website. For more information on this initiative please see our author guidelines.

Publication as an Article in Press is typically within 1-2 weeks after we have received your corrected proofs and publishing agreement. Subsequently, we will aim to publish the Version of Record in a timely manner. Please note there will be no further correspondence about your publication date.

When your article is published as the Version of Record, you will receive a notification email. **If you are planning an embargoed press release or require a specific publication date, please complete our scheduling requests form, or contact commsproduction@springernature.com, as soon as possible after acceptance and we will endeavour to accommodate your request.**

For further information on the journey of your article from acceptance to publication, please see our Author FAQs.

Publishing Agreements and Fees:

In about one week, you will receive an email with a link to complete the appropriate grant of rights necessary for publishing your paper and – if applicable – to provide payment information for your article-processing charge (APC), either via credit card or by requesting an invoice.

If needed, our Author Services team will be in touch regarding any additional information that may be required.

In order to avoid any delays, please ensure that you have emails from Springer Nature whitelisted in your mail system.

Proofs:

At this point we will also edit your manuscript to ensure that it conforms with our house style. Once you have completed the publication agreement and arranged payment, you will receive a separate email with a link to an online eProof for you to review. Please read your proof with great care to ensure that no changes have been introduced which have inadvertently altered the meaning of your paper. We suggest that you discuss the proof with your co-authors, but please ensure that only one author communicates with us and that only one set of corrections is returned via the online correction in the eProof.

The corresponding (or nominated) author is responsible on behalf of all co-authors for the accuracy of all content, including spelling of names and current affiliations.

To ensure prompt publication, your proofs should be returned within two working days. If there is any period within the next four weeks in which you won't be available, please nominate a co-author with whom we can correspond and send their contact information to us via email at commsproduction@springernature.com as soon as possible.

Please note that your Supplementary Information files are now finalised, and they will be submitted as provided for preparation for publication of the Article. Any requests to make changes will only be considered in exceptional circumstances and will result in a delay to publication.

You will not receive access to your eProof until the Licence to Publish and Article-Processing Charge steps are completed.

We welcome the submission of material for the ‘Featured Image’ section of the Communications Materials home page. Images should relate to the content of your manuscript, but do not need to be taken directly from the accepted work. Suggestions should be sent by email to commsmat@nature.com, along with the completed Licence to Publish form. Please note that images should be supplied as1400x400-pixel, in RGB. Unfortunately, we cannot promise that your suggestions will be used.

Providing great service is very important to us. We would greatly appreciate any comments you have about your experience at Communications Materials. We look forward to publishing your paper, and we hope to work with you again in the future.

Best regards,

John Plummer, PhD
Chief Editor
orcid.org/0000-0003-4824-8497
Communications Materials

*You can now use a single sign-on for all your accounts, view the status of all your manuscript submissions and reviews, access usage statistics for your published articles and download a record of your reviewing activity for the Nature Portfolio journals. Please check your account regularly and ensure that we have your current contact information.

** Visit Nature Research's author and referees' website at www.nature.com/authors for information about policies, services and author benefits**

We may promote your article on social media once it is published, so please feel free to send me the twitter handles of any authors or departments and we will be sure to tag them accordingly.

Open Access This Peer Review File is licensed under a Creative Commons Attribution 4.0 International License, which permits use, sharing, adaptation, distribution and reproduction in any medium or format, as long as you give appropriate credit to the original author(s) and the source, provide a link to the Creative Commons license, and indicate if changes were made.
In cases where reviewers are anonymous, credit should be given to 'Anonymous Referee' and the source.
The images or other third party material in this Peer Review File are included in the article's Creative Commons license, unless indicated otherwise in a credit line to the material. If material is not included in the article's Creative Commons license and your intended use is not permitted by statutory regulation or exceeds the permitted use, you will need to obtain permission directly from the copyright holder.
To view a copy of this license, visit https://creativecommons.org/licenses/by/4.0/

We are very grateful to all reviewers for their helpful comments which have improved the manuscript significantly. Our response to the points raised, and the changes made in the manuscript are outlined below (in blue text).

Reviewer 1

Key Questions:

- After examination of Figures 3, S1 and S2, we wonder whether there could have been undetectable non-equatorial buckling before the equatorial buckling appears at 16 % ? Especially, does the possibility exist that buckling appears at a high mode associated with too small deflections to be visualized, and then a mode switch towards equatorial buckling would happen ? Have you tried to build reconstructions at several deformations ε but in relative rcurv scales rather than an absolute scale ?

The reviewer is correct that we cannot be 100% sure that there are not earlier phenomena below our detection limit, which is unfortunately a concern in all imaging endeavours. However, the theories outlined in the works we reference (Jones et al.) do not predict non-equatorial buckling for these materials whereas the buckling observed here displays wavelength trends relative to δ and ζ that qualitatively match expectation from theory. Additionally, we do not see any earlier-mode deformations in our macro experiment, where we are not as close to resolution limit concerns. We have added a note into the manuscript noting the detection limit concern "The images at low strain (ε = 8%) show that the equatorial buckling observed is not due to a pre-existing defect and. is very likely the shell instability event for these microspheres under uniaxial compression. The possibility of an earlier non-equatorial buckling pattern below our resolution limit cannot be excluded, but this is not theoretically predicted, nor observed in the macroscale experiment." We attempted relative rcurv scales during the initial analysis however as there are always regions of very high curvature somewhere in the scans, due to shell defects and/or surface generation errors (which can vary from scan-to-scan, see 20% strain example below), any automatic or relative scaling tends to wash out the buckling visualisation. The below visualisation shows the same spheres at the same relative *proportions* of the full curvature range (for each strain). We find imposing a fixed r_curv range at the lower end of the curvature range better highlights the buckling development.

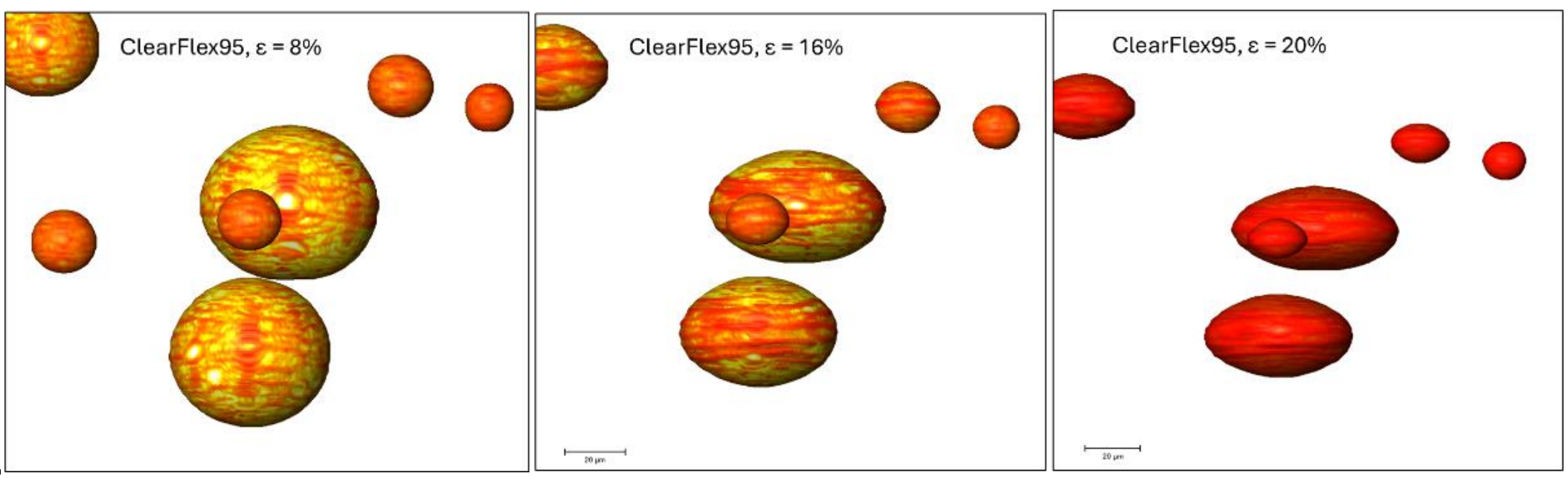

- At moderate strain (i.e. 8%) for the smallest spheres where the all-over buckling regime is approached, the buckling pattern should extend to the whole sphere, theoretically in “longitude fringes” as I understand. However, in addition to these “longitude fringes”, there seems to be “latitude fringes” on Figures 3, S1 and S2. Is that the case ? If so, how do you define the buckling wavelength?

The latitude fringes are an artefact of the surface generation from the segmented image data. This is clear when looking at the 0% strain images, where these are still present (which is why some 0% images are provided in the supplementary). These artefacts also mean the fixed rcurv scale is better for showing the development of the buckling pattern – which completely replaces the latitude fringes at higher strain. We have added a note into the manuscript at the point Figure 3 is introduced to describe these artefacts. “The vertical fringes visible in Figure 3(c) (highlighted by the grey arrow) are artefacts of the surface generation process (described in the Methods), rather than a form of buckling. This is clear when looking at ε = 0% images, where these fringes are still present; see Figures S3-5 in the Supplementary Information.”

- Does the critical strain observed experimentally at buckling onset match the one predicted by the model of Jones et al. [27]? If not, what is the possible explanation? Could it be in connection with the precedent question? In fact, it is practically difficult to relate the colormap to specific values in Fig. 5(a).

Unfortunately, we cannot measure the critical buckling strain from our images due to the finite resolution of the techniques. We have updated our comments on this point within the manuscript “It is not possible to quantify a value for the critical buckling strain from the experimental data, due to the resolution of the imaging techniques. To observe buckling, the microspheres must first be strained by an unknown quantity beyond the onset of buckling until the amplitude of buckling can be resolved. This is not an issue for the buckling wavelength measurement however, as only the amplitude is expected to increase with strain increases beyond the onset of initial buckling.” We agree with the reviewer regarding Figure 5 and have added contours onto (a) and (b) to aid the relation of the colours.

- Finally, a recent article (which the authors could not have known about at the time of writing) by Madeira et al. (https://doi.org/10.1016/j.matdes.2025.115234) provides an analysis of the pre- and post-buckling of HTMs under hydrostatic compression. This reference should be cited, the discussion of the present paper may benefit from a comparison with some results of this paper.

We thank the reviewer for highlighting this impressive paper. The microspheres examined are the same class as ours but the compression is hydrostatic. As such we cannot make any meaningful comparison between the pre- and post-buckling states in these two papers. We have cited this paper when discussing previous works that

have examined microsphere buckling however, in the introduction: “Pre- and post-buckling states of hollow thermoplastic microspheres was recently visualised for syntactic foams under hydrostatic loading however (where different buckling modes are expected) [33].”. The phrase uniaxial compression has been added in the preceding paragraph for clarity.

Detailed remarks:

1. The title is vague. It should specify ‘uniaxial compression’. In addition, it would be more precise to mention what the microspheres are “embedded” into: ‘into an elastomer’? ‘into an incompressible matrix’? ‘into a rubber-like material’?... Moreover, the nature of the microspheres (HTMs) could be given.

We have updated the title to “Buckling of elastomer-embedded thermoplastic microspheres under uniaxial compression revealed by in-situ X-ray micro-CT”

2. In the abstract, ‘under uniaxial compression’ should be specified.

We have added this clarification “Here we demonstrate, in-situ, the buckling of embedded thermoplastic shells under uniaxial compression”

3. In the abstract, should we refer to ‘absolute size’? In the text, the authors refer instead to the ‘shell wall thickness ratio’, which is a dimensionless quantity.

We have adjusted the sentence to be clearer and more in line with the main text… “The results validate several important predictions from theory; regardless of sphere size, equatorial buckling prevails whenever the shell walls are thin relative to their diameter.”

4. Line 43: we do not know if Fig. 1(c-d) refers to tension or compression. Specify in the text and in the caption of the figure.

We have added notes into the text and caption to clarify that they are loaded in compression.

5. In the caption of Figure 1, the elastomer matrix should be specified.

We have changed “polymer-matrix” to “polyurethane-matrix” in the caption

6. Line 75: specify ‘small uniaxial compression...’.

We have changed the line to read “Theoretical investigations into the buckling of a single embedded shell under small uniaxial, small strain compressive loading"

7. The paragraph between lines 82 and 92 mentions a number of results concerning the buckling phenomenon and the corresponding buckling patterns. This should be supported by bibliographical references.

We have added the relevant references, which are from Jones et al. cited elsewhere but were missed from this paragraph. We have also changed some statements to “should” statements to reflect better that these are predictions.

8. Line 92: the authors claim that the wavelength of buckling patterns is comparable to shell radius. Are you sure of this order of magnitude? As an example, Hutchinson (Proc R Soc London A, 2016, at the end of page 9) mention that 'the deflections are of the order of several shell thicknesses'. This should be clarified.

We believe that Hutchinson is referring to the amplitude of the buckling when mentioning "deflections", whereas we are discussing the wavelength of buckling. We also believe the work mentioned is for hydrostatic compression, whereas we are discussing uniaxial loading.

9. In Table 1, specify that ClearFlex is a polyurethane (even though this is mentioned in line 147).

We have added this clarification into the table

10. Line 148: specify the strain rate (in s-1) rather than the loading rate (in mm/min).

We have added the strain rate, in s^{-1} alongside the engineering loading rate

11. Similarly, strain rates should be indicated for 'micro-scale experiments' (lines 172-175).

We have added this information, and re-worded the surrounding sentences to accommodate, also directing the reader to the loading/imaging scheme figure in the methods section "Compressive loading was incremental in small steps from 0% strain to a level considerably beyond the onset of buckling (up to 20-35%) using a loading speed used of 0.02 mm/min (equivalent to a strain rate of 2.78 x 10-5 s-1). with a A full tomogram (pixel size 0.65 μm) was collected at each numerous static strains, during both loading and unloading of each sample (see Methods section), allowing the first onset of buckling to be imaged."

12. Lines 186–187: We do not understand why 'the images show that the buckling is not due to a pre-existing defect'. Is it related to the black arrow in Fig. 3? This argument needs to be justified.

The lack of any visible defect on the central microsphere indicates that the buckling pattern which ultimately emerges is not due to any detectable defect. Furthermore, even when off-axis defects *is* present (on a nearby sphere - the black arrow example), the equatorial buckling pattern emerges regardless. We have re-worked this section for clarity (and moved below the comments on detection limit) "The images at low strain (ε = 8%) also show that the equatorial buckling observed is not due to a pre-existing defect, as none are present for the central microsphere in the images. Furthermore, as highlighted by the black arrow in Figure3(a,b), the buckling pattern emerges in a nearby microsphere despite an off-equator defect, showing that the equatorial buckling is a robust effect."

13. Line 193: looking at figure S2 at 8%, small waves can be seen on the spheres (fig. a)). How do the authors differentiate between these waves and those in figure b)?

Perhaps they are identical, but with a lower amplitude? Please note that there is a problem in the caption for figure S2: b) vs c).

These are likely the same buckling fringes, but at lower amplitude, as the reviewer says. This is likely result of the microsphere selected being particularly large. Although not intentional, we suggest keeping the figure as it is, as it gives an impression of the earlier buckling that is *predicted* for larger microspheres. Although we have removed this claim from the main text (as per comment #15 below), we have updated the caption to allow the reader to make their own judgement on this. "Some evidence of buckling Is visible here at the lower strain, which may be attributable to this sphere being particularly large (diameter = 205 μm) which, according to predictions from theory, should lead to a lower critical buckling wavelength (assuming an unchanged shell wall thickness)". We have also fixed the caption issue b) vs c).

14. Line 213-216: for key prediction i), it should be precised that h has to be considered constant (in relation with authors' work [23]). Otherwise, if h varies, the wall ratio could remain constant.

We thank the reviewer for identifying this omission. We have added some explanation into the text "Note: for trend i) we assume a fixed shell wall thickness for all microspheres, such that the trend can be assessed in terms of the microsphere diameter only, rather than the parameter $\zeta = h/R$. Previous measurements of Expancel 920 microspheres revealed an average shell wall thickness of ~290 nm, with no dependence on the microsphere diameter [23]."

15. Once again, to discuss the spheres that buckled and those that did not (lines 270-272), would it be possible to find a graphical way (or create other figures) to show which spheres are buckled in Figures S3-S5?

We have attempted to create new figures to demonstrate this however it is very difficult to convincingly visualise. This is because close the critical buckling strain the amplitude of buckling can be close to or below the detection limit of the techniques in this experiment. We are less convinced of this claim now, so have removed it from the text. We instead remain focussed on the buckling wavelength visualisation and trends in ζ and/or δ, which were the principal aims of this paper. We do however leave a related remark within the caption of Figure S2, as described above.

16. Lines 289-292: the authors invoke viscoelasticity in the discussion. For this remark to be relevant, it would be necessary to discuss the order of magnitude of the strain rate and to specify the rate at which viscoelasticity must be taken into account.

For points #10 and #11 above we have added strain rate information into the manuscript. Given the low strain rates here (~10^{-5} s^{-1}) we do not expect any viscoelastic effects. We have added a relevant reference for this which can be used to calculate a relaxation time for our conditions (approx. 2-3 s – very quick compared to

our loading rate and hold times given in the methods). We have added a note on this into the text, alongside the re-wording of the final paragraphs, that clarifies viscoelasticity may be a concern for other, higher-strain, applications of similar microsphere-containing materials.

17. In our opinion, the last paragraph of the discussion (lines 294-316) weakens it. It would be relevant to reduce it to the essentials and include it in the introduction instead.

We agree with the reviewer and have significantly shorterned/re-worked the final paragraph – only giving a brief impression of possible future applications of the buckling pattern control.

18. Line 480: the authors mention the use of a Gent model to determine the 'infinitesimal shear modulus'. In fact, for small strains, this model reduces to the neo-Hookean model; it is therefore the latter that has been fitted. It would be more accurate not to mention the Gent model in this context.

The reason for fitting the stress-strain curve was that the data from the Instron machine was not especially clean, especially at low strains. When trying to fit a straight line to this data at very low strains we would therefore end up with a wide variation in the elastic modulus for each sample despite the stress-strain curves otherwise appearing very similar. Extending the fitting region further introduces its own biases as the limits of this region woud be arbitrary. To account for this in an objective way it was decided to fit a nonlinear model to the whole stress-strain curve, and extract the infinitesimal modulus from the nonlinear model. We have added a little more information into the methods at the relevant point to explain this "Due to noise in the data, from the testing equipment, at low strains, Aa Gent model was fitted to this data to extract the infinitesimal shear modulus."

19. Line 502: We cannot find the value vs = 0.43 in reference [23]. Should it not be 0.23 instead (Tab. 3 in [23])?

0.43 is correct for the macroexperiment sphere, but we have incorrectly referenced towards the microexperiment spheres. We have replaced with the correct reference for the macroexperiment .

20. In Figure M1, it should be specified whether the 'true' or 'nominal' stress is used for the y-axis.

We have clarified this within the caption as the nominal stress.

Reviewer 3

This paper focuses on the compressive buckling behavior of embedded microspheres

in syntactic foams, verifying the theoretically predicted equatorial buckling mode through multi-scale in-situ experiments. It clarifies the correlation between buckling behavior and dimensionless parameters ζ (shell thickness/radius) and δ (matrix/shell shear modulus ratio), providing critical experimental support for regulating the mechanical properties of composite materials. The research topic holds theoretical value and application prospects. However, before recommending this manuscript for publication, the authors should address the following points to strengthen its rigor and clarity.

1. In Table 2, the measured normalized buckling wavelengths of shells in Silicone and ClearFlex50 matrices are notably lower than the theoretical predictions, with good agreement only observed for the ClearFlex95 matrix. The paper attributes this discrepancy to "the linear elasticity assumption being more valid in rigid matrices," which lacks adequate rigor. As illustrated by the stress-strain curve of the ClearFlex50-matrix composite (Figure M2(b)), the material exhibits excellent elastic linearity within the tested strain range. The authors should discuss additional potential factors to strengthen the rationality of the explanation.

Linear stability provides an indication of the likely instabilities and the trends that are expected to develop when parameters are modified. We agree that it is perhaps not helpful to speculate on the possible reasons why the numerical values are not precise. However, we do note that the stability analysis agrees with the expected trends when varying parameters. We have removed this remark from the paper and simply highlight the discrepancy and leave it as a question for the reader. "At present it is not known why there is a discrepancy in the measured and theoretical values of ƛ for the other two matrices but, as noted previously, the trends with respect to ζ and δ appear valid."

2. The paper does not clarify the interface state between the shells and the matrix during buckling (e.g., perfect bonding, partial debonding, or slip). It remains unclear whether interface strength or bonding quality affects the initiation of buckling, the evolution of buckling mode, or the critical buckling strain. Supplementary characterizations of the shell-matrix interface are recommended to clarify the role of interface in buckling behavior.

In the microscale experiments the shell wall itself cannot be resolved, as it is thin (~290nm) relative to the spatial resolution (~1.95 μm) and similar in density to the matrix. The position and shape of the microspheres is revealed by X-ray CT due to the large density differences between the matrix material and air within the microspheres. We are assuming good bonding on the basis that we detect a buckling pattern, i.e. the shell-wall/matrix interface must be maintained during compression to give the observed ripples. If debonding had occurred, we would instead image more regular compressed ellipsoid shape as the microsphere would then act like a cavity (with de-bonded shell material within). We cannot rule out partial debonding of the

microspheres however and have noted this and the above in the paper. “The microsphere shells are thin and similar in density to the matrix material and therefore cannot be resolved directly from these X-ray CT images; whereas the microsphere interior (air) provides good contrast. The detection of the buckling pattern implies the matrix-shell interface remains relatively intact (otherwise the microsphere would act as a cavity and the X-ray CT images would show a regular compressed ellipsoid shape). The possibility of partial debonding cannot be excluded however, which may explain the slight mismatch between the theoretical and measured buckling wavelengths (discussed later).” In the case of the macroscale experiment no debonding is observed and we now note this in the text “We do not observe any indication of matrix-shell debonding, even at high strains (~20%).”

3. According to the theoretical framework, the buckling modes (equatorial buckling and all-over buckling) are governed by the dimensionless parameters ζ and δ. In Figure 5, some experimental data points lie close to the theoretical boundary between the two buckling modes, yet the shells consistently exhibit typical equatorial buckling characteristics. This raises questions about the accuracy of the theoretical boundary or the influence of unaccounted factors in the experiments.

We thank the reviewer for highlighting this mistake. Non-equatorial buckling is expected towards the bottom right corner of our plots (high ζ, low δ). However, in our submission, we wrongly labelled a strain contour in this area as the boundary for all-over vs equatorial buckling. We have corrected this, alongside adding new contours for readability as requested previously. As ζ increases and/or δ decreases we would expect all-over buckling to become more prevalent but the exact parameter boundaries are unknown, so we have removed this label.

4. The authors emphasize that the nonlinear response of syntactic foams can be modulated by tuning the dimensionless parameters ζ and δ, and further propose potential applications in soft robotics and strain sensing. However, the manuscript lacks quantitative design schemes that bridge theoretical parameters, material properties, and target application performance, leaving a gap between basic research findings and practical engineering applications.

We have significantly restructured the final discussion to give less importance to these applications (also in response to Reviewer1 points #16, #17). We mention the possibility of tuning wavelength via those parameters but have removed all mention of soft robotics from the paper. We now only propose strain sensing in the abstract and briefly mention optical usages as possible avenues for exploration in the discussion. We feel any quantitative design schemes are beyond the scope of this paper. It is unlikely that the specific matrix materials and microspheres we have studied would be suitable for the proposed applications, but the tunability of shell buckling wavelength *in general* is worthy of exploration.

5. Figure 4 scales microspheres of different sizes to the same dimensions for comparative convenience, but this conceals the intuitive impact of actual size differences on buckling wavelength. The authors are advised to clearly indicate the scaling ratio in the legend to avoid misinterpretation by the readers.

The scaling ratio is different for each sphere, so a single value cannot be given in the legend. We have instead replaced the indicative scale bars for each size class with a scalebar for each individual sphere. Regardless of size, the scalebar is 30µm which gives a quick indication of the microsphere true size.

6. This paper qualitatively depicts buckling regions via curvature color mapping but lacks quantitative curvature data. The authors are suggested to plot curves of curvature changes with strain to quantify the evolution of buckling degree.

The r_curv colour mapping was only used to highlight the buckling pattern for 2D print/screen format. Quantified values of the microsphere curvature will not describe buckling wavelength, which is our main focus here. The buckling wavelengths reported were interpreted from the *spacing* of ripples on the surface renderings, i.e. the *spacing* of the bands of high curvature. The values of r_curv in those bands might be related, in some way, to the amplitude of the buckling but this is not of less interest as we do not know, on a sphere-to-sphere basis, how far beyond the true critical buckling strain we are (as now mentioned in the paper, following reviewer 1's comments). We feel this makes any meaningful interpretation of r_curv difficult, and beyond the scope of this paper. We have added more discussion of the meaning of r_curv into the manuscript "These colour mappings are provided to highlight areas where the microsphere deviates from an ellipsoidal shape, i.e. where the shell has buckled." And "Values of rcurv at different strain levels or for different microsphere sizes are not provided here as the colour mappings (Figures 3–5, S1-5) are given simply to better highlight the buckling pattern".

7. A recent study on syntactic foam failure behaviors (European Journal of Mechanics-A/Solids, 2024, 105: 105256) aligns closely with the research theme and is recommended for appropriate citation.

We thank the reviewer for highlighting this impressive paper. Although it is focussed on hydrostatic loading, whereas we focus on uniaxial, it still provides excellent context for microstructure-property relations for microsphere composites. We have cited this paper several times in the introduction

8. This paper assumes all matrices have a Poisson's ratio of 0.49 (based on the incompressible assumption), while no experimental basis or justification is provided for this assumption.

The Poisson's ratio for elastomers is generally close to 0.5, and we have added a reference into the paper to support this. We have assumed 0.49 based some references based on experimental work on similar materials, which we have also referenced.

Response to Reviewers

We are very grateful to all reviewers for their helpful comments which have improved the manuscript significantly. Our response to the points raised, and the changes made in the manuscript are outlined below (in blue text). We have split this document into two, one for each round of reviewer comments.

Revisions #2

Reviewer 3

Since the authors adopted the term of 'Buckling ...' in the title, do we suppose to see a fully recoverable configuration for those spherical shells? If not, words like 'collapse', 'damage', 'failure' could be more precise.

We do expect reversible deformation of the shells after loading/unloading, which we believe is the origin of their recoverable mechanical performance under straining. Conversely, syntactic foams containing glass microspheres, which may fracture under loading, exhibit poor recoverability. This is discussed in the introduction " Therefore we are happy with the term buckling and would prefer this to collapse, etc.

Revisions #1

Reviewer 1

Key Questions:

- After examination of Figures 3, S1 and S2, we wonder whether there could have been undetectable non-equatorial buckling before the equatorial buckling appears at 16 % ? Especially, does the possibility exist that buckling appears at a high mode associated with too small deflections to be visualized, and then a mode switch towards equatorial buckling would happen ? Have you tried to build reconstructions at several deformations ε but in relative rcurv scales rather than an absolute scale ?

The reviewer is correct that we cannot be 100% sure that there are not earlier phenomena below our detection limit, which is unfortunately a concern in all imaging endeavours. However, the theories outlined in the works we reference (Jones et al.) do not predict non-equatorial buckling for these materials whereas the buckling observed here displays wavelength trends relative to δ and ζ that qualitatively match expectation from theory. Additionally, we do not see any earlier-mode deformations in our macro experiment, where we are not as close to resolution limit concerns. We

have added a note into the manuscript noting the detection limit concern “The images at low strain (ε = 8%) show that the equatorial buckling observed is not due to a pre-existing defect and. is very likely the shell instability event for these microspheres under uniaxial compression. The possibility of an earlier non-equatorial buckling pattern below our resolution limit cannot be excluded, but this is not theoretically predicted, nor observed in the macroscale experiment.” We attempted relative rcurv scales during the initial analysis however as there are always regions of very high curvature somewhere in the scans, due to shell defects and/or surface generation errors (which can vary from scan-to-scan, see 20% strain example below), any automatic or relative scaling tends to wash out the buckling visualisation. The below visualisation shows the same spheres at the same relative *proportions* of the full curvature range (for each strain). We find imposing a fixed r_curv range at the lower end of the curvature range better highlights the buckling development.

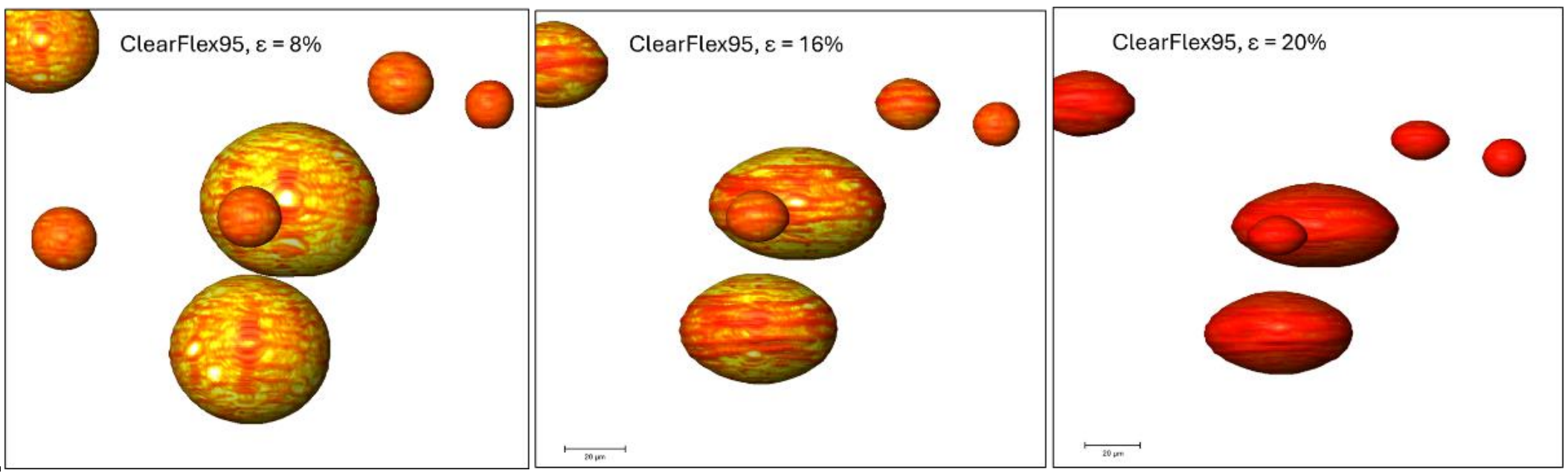

- At moderate strain (i.e. 8%) for the smallest spheres where the all-over buckling regime is approached, the buckling pattern should extend to the whole sphere, theoretically in “longitude fringes” as I understand. However, in addition to these “longitude fringes”, there seems to be “latitude fringes” on Figures 3, S1 and S2. Is that the case ? If so, how do you define the buckling wavelength?

The latitude fringes are an artefact of the surface generation from the segmented image data. This is clear when looking at the 0% strain images, where these are still present (which is why some 0% images are provided in the supplementary). These artefacts also mean the fixed rcurv scale is better for showing the development of the buckling pattern – which completely replaces the latitude fringes at higher strain. We have added a note into the manuscript at the point Figure 3 is introduced to describe these artefacts. “The vertical fringes visible in Figure 3(c) (highlighted by the grey arrow) are artefacts of the surface generation process (described in the Methods), rather than a form of buckling. This is clear when looking at ε = 0% images, where these fringes are still present; see Figures S3-5 in the Supplementary Information.”

- Does the critical strain observed experimentally at buckling onset match the one predicted by the model of Jones et al. [27]? If not, what is the possible explanation? Could it be in connection with the precedent question? In fact, it is practically difficult to relate the colormap to specific values in Fig. 5(a).

Unfortunately, we cannot measure the critical buckling strain from our images due to the finite resolution of the techniques. We have updated our comments on this point within the manuscript "It is not possible to quantify a value for the critical buckling strain from the experimental data, due to the resolution of the imaging techniques. To observe buckling, the microspheres must first be strained by an unknown quantity beyond the onset of buckling until the amplitude of buckling can be resolved. This is not an issue for the buckling wavelength measurement however, as only the amplitude is expected to increase with strain increases beyond the onset of initial buckling." We agree with the reviewer regarding Figure 5 and have added contours onto (a) and (b) to aid the relation of the colours.

- Finally, a recent article (which the authors could not have known about at the time of writing) by Madeira et al. (https://doi.org/10.1016/j.matdes.2025.115234) provides an analysis of the pre- and post-buckling of HTMs under hydrostatic compression. This reference should be cited, the discussion of the present paper may benefit from a comparison with some results of this paper.

We thank the reviewer for highlighting this impressive paper. The microspheres examined are the same class as ours but the compression is hydrostatic. As such we cannot make any meaningful comparison between the pre- and post-buckling states in these two papers. We have cited this paper when discussing previous works that have examined microsphere buckling however, in the introduction: "Pre- and post-buckling states of hollow thermoplastic microspheres was recently visualised for syntactic foams under hydrostatic loading however (where different buckling modes are expected) [33].". The phrase uniaxial compression has been added in the preceding paragraph for clarity.

Detailed remarks:

1. The title is vague. It should specify 'uniaxial compression'. In addition, it would be more precise to mention what the microspheres are "embedded" into: 'into an elastomer'? 'into an incompressible matrix'? 'into a rubber-like material'?... Moreover, the nature of the microspheres (HTMs) could be given.

We have updated the title to "Buckling of elastomer-embedded thermoplastic microspheres under uniaxial compression revealed by in-situ X-ray micro-CT"

2. In the abstract, 'under uniaxial compression' should be specified.

We have added this clarification "Here we demonstrate, in-situ, the buckling of embedded thermoplastic shells under uniaxial compression"

3. In the abstract, should we refer to 'absolute size'? In the text, the authors refer instead to the 'shell wall thickness ratio', which is a dimensionless quantity.

We have adjusted the sentence to be clearer and more in line with the main text… "The results validate several important predictions from theory; regardless of sphere size, equatorial buckling prevails whenever the shell walls are thin relative to their diameter."

4. Line 43: we do not know if Fig. 1(c-d) refers to tension or compression. Specify in the text and in the caption of the figure.

We have added notes into the text and caption to clarify that they are loaded in compression.

5. In the caption of Figure 1, the elastomer matrix should be specified.

We have changed "polymer-matrix" to "polyurethane-matrix" in the caption

6. Line 75: specify 'small uniaxial compression...'.

We have changed the line to read "Theoretical investigations into the buckling of a single embedded shell under small uniaxial, small strain compressive loading"

7. The paragraph between lines 82 and 92 mentions a number of results concerning the buckling phenomenon and the corresponding buckling patterns. This should be supported by bibliographical references.

We have added the relevant references, which are from Jones et al. cited elsewhere but were missed from this paragraph. We have also changed some statements to "should" statements to reflect better that these are predictions.

8. Line 92: the authors claim that the wavelength of buckling patterns is comparable to shell radius. Are you sure of this order of magnitude? As an example, Hutchinson (Proc R Soc London A, 2016, at the end of page 9) mention that 'the deflections are of the order of several shell thicknesses'. This should be clarified.

We believe that Hutchinson is referring to the amplitude of the buckling when mentioning "deflections", whereas we are discussing the wavelength of buckling. We also believe the work mentioned is for hydrostatic compression, whereas we are discussing uniaxial loading.

9. In Table 1, specify that ClearFlex is a polyurethane (even though this is mentioned in line 147).

We have added this clarification into the table

10. Line 148: specify the strain rate (in s-1) rather than the loading rate (in mm/min).

We have added the strain rate, in s^{-1} alongside the engineering loading rate

11. Similarly, strain rates should be indicated for 'micro-scale experiments' (lines 172-175).

We have added this information, and re-worded the surrounding sentences to accommodate, also directing the reader to the loading/imaging scheme figure in the methods section "Compressive loading was incremental in small steps from 0% strain to a level considerably beyond the onset of buckling (up to 20-35%) using a loading speed used of 0.02 mm/min (equivalent to a strain rate of 2.78 x 10-5 s-1). with a A full tomogram (pixel size 0.65 μm) was collected at each numerous static strains, during both loading and unloading of each sample (see Methods section), allowing the first onset of buckling to be imaged."

12. Lines 186–187: We do not understand why 'the images show that the buckling is not due to a pre-existing defect'. Is it related to the black arrow in Fig. 3? This argument needs to be justified.

The lack of any visible defect on the central microsphere indicates that the buckling pattern which ultimately emerges is not due to any detectable defect. Furthermore, even when off-axis defects *is* present (on a nearby sphere - the black arrow example), the equatorial buckling pattern emerges regardless. We have re-worked this section for clarity (and moved below the comments on detection limit) "The images at low strain (ε = 8%) also show that the equatorial buckling observed is not due to a pre-existing defect, as none are present for the central microsphere in the images. Furthermore, as highlighted by the black arrow in Figure3(a,b), the buckling pattern emerges in a nearby microsphere despite an off-equator defect, showing that the equatorial buckling is a robust effect."

13. Line 193: looking at figure S2 at 8%, small waves can be seen on the spheres (fig. a)). How do the authors differentiate between these waves and those in figure b)? Perhaps they are identical, but with a lower amplitude? Please note that there is a problem in the caption for figure S2: b) vs c).

These are likely the same buckling fringes, but at lower amplitude, as the reviewer says. This is likely result of the microsphere selected being particularly large. Although not intentional, we suggest keeping the figure as it is, as it gives an impression of the earlier buckling that is *predicted* for larger microspheres. Although we have removed this claim from the main text (as per comment #15 below), we have updated the caption to allow the reader to make their own judgement on this. "Some evidence of buckling Is visible here at the lower strain, which may be attributable to this sphere being particularly large (diameter = 205 μm) which, according to predictions from theory, should lead to a lower critical buckling wavelength (assuming an unchanged shell wall thickness)". We have also fixed the caption issue b) vs c).

14. Line 213-216: for key prediction i), it should be precised that h has to be considered constant (in relation with authors' work [23]). Otherwise, if h varies, the wall ratio could remain constant.

We thank the reviewer for identifying this omission. We have added some explanation into the text "Note: for trend i) we assume a fixed shell wall thickness for all

microspheres, such that the trend can be assessed in terms of the microsphere diameter only, rather than the parameter $\zeta = h/R$. Previous measurements of Expancel 920 microspheres revealed an average shell wall thickness of ~290 nm, with no dependence on the microsphere diameter [23]."

15. Once again, to discuss the spheres that buckled and those that did not (lines 270-272), would it be possible to find a graphical way (or create other figures) to show which spheres are buckled in Figures S3-S5?

We have attempted to create new figures to demonstrate this however it is very difficult to convincingly visualise. This is because close the critical buckling strain the amplitude of buckling can be close to or below the detection limit of the techniques in this experiment. We are less convinced of this claim now, so have removed it from the text. We instead remain focussed on the buckling wavelength visualisation and trends in ζ and/or δ, which were the principal aims of this paper. We do however leave a related remark within the caption of Figure S2, as described above.

16. Lines 289-292: the authors invoke viscoelasticity in the discussion. For this remark to be relevant, it would be necessary to discuss the order of magnitude of the strain rate and to specify the rate at which viscoelasticity must be taken into account.

For points #10 and #11 above we have added strain rate information into the manuscript. Given the low strain rates here (~10^{-5} s^{-1}) we do not expect any viscoelastic effects. We have added a relevant reference for this which can be used to calculate a relaxation time for our conditions (approx. 2-3 s – very quick compared to our loading rate and hold times given in the methods). We have added a note on this into the text, alongside the re-wording of the final paragraphs, that clarifies viscoelasticity may be a concern for other, higher-strain, applications of similar microsphere-containing materials.

17. In our opinion, the last paragraph of the discussion (lines 294-316) weakens it. It would be relevant to reduce it to the essentials and include it in the introduction instead.

We agree with the reviewer and have significantly shorterned/re-worked the final paragraph – only giving a brief impression of possible future applications of the buckling pattern control.

18. Line 480: the authors mention the use of a Gent model to determine the 'infinitesimal shear modulus'. In fact, for small strains, this model reduces to the neo-Hookean model; it is therefore the latter that has been fitted. It would be more accurate not to mention the Gent model in this context.

The reason for fitting the stress-strain curve was that the data from the Instron machine was not especially clean, especially at low strains. When trying to fit a straight line to this data at very low strains we would therefore end up with a wide variation in the

elastic modulus for each sample despite the stress-strain curves otherwise appearing very similar. Extending the fitting region further introduces its own biases as the limits of this region woud be arbitrary. To account for this in an objective way it was decided to fit a nonlinear model to the whole stress-strain curve, and extract the infinitesimal modulus from the nonlinear model. We have added a little more information into the methods at the relevant point to explain this "Due to noise in the data, from the testing equipment, at low strains, Aa Gent model was fitted to this data to extract the infinitesimal shear modulus."

19. Line 502: We cannot find the value vs = 0.43 in reference [23]. Should it not be 0.23 instead (Tab. 3 in [23])?

0.43 is correct for the macroexperiment sphere, but we have incorrectly referenced towards the microexperiment spheres. We have replaced with the correct reference for the macroexperiment .

20. In Figure M1, it should be specified whether the 'true' or 'nominal' stress is used for the y-axis.

We have clarified this within the caption as the nominal stress.

Reviewer 3

This paper focuses on the compressive buckling behavior of embedded microspheres in syntactic foams, verifying the theoretically predicted equatorial buckling mode through multi-scale in-situ experiments. It clarifies the correlation between buckling behavior and dimensionless parameters ζ (shell thickness/radius) and δ (matrix/shell shear modulus ratio), providing critical experimental support for regulating the mechanical properties of composite materials. The research topic holds theoretical value and application prospects. However, before recommending this manuscript for publication, the authors should address the following points to strengthen its rigor and clarity.

1. In Table 2, the measured normalized buckling wavelengths of shells in Silicone and ClearFlex50 matrices are notably lower than the theoretical predictions, with good agreement only observed for the ClearFlex95 matrix. The paper attributes this discrepancy to "the linear elasticity assumption being more valid in rigid matrices," which lacks adequate rigor. As illustrated by the stress-strain curve of the ClearFlex50-matrix composite (Figure M2(b)), the material exhibits excellent elastic linearity within the tested strain range. The authors should discuss additional potential factors to strengthen the rationality of the explanation.

Linear stability provides an indication of the likely instabilities and the trends that are expected to develop when parameters are modified. We agree that it is perhaps not helpful to speculate on the possible reasons why the numerical values are not precise. However, we do note that the stability analysis agrees with the expected trends when varying parameters. We have removed this remark from the paper and simply highlight the discrepancy and leave it as a question for the reader. “At present it is not known why there is a discrepancy in the measured and theoretical values of λ for the other two matrices but, as noted previously, the trends with respect to ζ and δ appear valid.”

2. The paper does not clarify the interface state between the shells and the matrix during buckling (e.g., perfect bonding, partial debonding, or slip). It remains unclear whether interface strength or bonding quality affects the initiation of buckling, the evolution of buckling mode, or the critical buckling strain. Supplementary characterizations of the shell-matrix interface are recommended to clarify the role of interface in buckling behavior.

In the microscale experiments the shell wall itself cannot be resolved, as it is thin (~290nm) relative to the spatial resolution (~1.95 μm) and similar in density to the matrix. The position and shape of the microspheres is revealed by X-ray CT due to the large density differences between the matrix material and air within the microspheres. We are assuming good bonding on the basis that we detect a buckling pattern, i.e. the shell-wall/matrix interface must be maintained during compression to give the observed ripples. If debonding had occurred, we would instead image more regular compressed ellipsoid shape as the microsphere would then act like a cavity (with de-bonded shell material within). We cannot rule out partial debonding of the microspheres however and have noted this and the above in the paper. “The microsphere shells are thin and similar in density to the matrix material and therefore cannot be resolved directly from these X-ray CT images; whereas the microsphere interior (air) provides good contrast. The detection of the buckling pattern implies the matrix-shell interface remains relatively intact (otherwise the microsphere would act as a cavity and the X-ray CT images would show a regular compressed ellipsoid shape). The possibility of partial debonding cannot be excluded however, which may explain the slight mismatch between the theoretical and measured buckling wavelengths (discussed later).” In the case of the macroscale experiment no debonding is observed and we now note this in the text “We do not observe any indication of matrix-shell debonding, even at high strains (~20%).”

3. According to the theoretical framework, the buckling modes (equatorial buckling and all-over buckling) are governed by the dimensionless parameters ζ and δ. In Figure 5, some experimental data points lie close to the theoretical boundary between the two buckling modes, yet the shells consistently exhibit typical equatorial buckling characteristics. This raises questions about the accuracy of the theoretical boundary or the influence of unaccounted factors in the experiments.

We thank the reviewer for highlighting this mistake. Non-equatorial buckling is expected towards the bottom right corner of our plots (high ζ, low δ). However, in our submission, we wrongly labelled a strain contour in this area as the boundary for all-over vs equatorial buckling. We have corrected this, alongside adding new contours for readability as requested previously. As ζ increases and/or δ decreases we would expect all-over buckling to become more prevalent but the exact parameter boundaries are unknown, so we have removed this label.

4. The authors emphasize that the nonlinear response of syntactic foams can be modulated by tuning the dimensionless parameters ζ and δ, and further propose potential applications in soft robotics and strain sensing. However, the manuscript lacks quantitative design schemes that bridge theoretical parameters, material properties, and target application performance, leaving a gap between basic research findings and practical engineering applications.

We have significantly restructured the final discussion to give less importance to these applications (also in response to Reviewer1 points #16, #17). We mention the possibility of tuning wavelength via those parameters but have removed all mention of soft robotics from the paper. We now only propose strain sensing in the abstract and briefly mention optical usages as possible avenues for exploration in the discussion. We feel any quantitative design schemes are beyond the scope of this paper. It is unlikely that the specific matrix materials and microspheres we have studied would be suitable for the proposed applications, but the tunability of shell buckling wavelength *in general* is worthy of exploration.

5. Figure 4 scales microspheres of different sizes to the same dimensions for comparative convenience, but this conceals the intuitive impact of actual size differences on buckling wavelength. The authors are advised to clearly indicate the scaling ratio in the legend to avoid misinterpretation by the readers.

The scaling ratio is different for each sphere, so a single value cannot be given in the legend. We have instead replaced the indicative scale bars for each size class with a scalebar for each individual sphere. Regardless of size, the scalebar is 30μm which gives a quick indication of the microsphere true size.

6. This paper qualitatively depicts buckling regions via curvature color mapping but lacks quantitative curvature data. The authors are suggested to plot curves of curvature changes with strain to quantify the evolution of buckling degree.

The r_curv colour mapping was only used to highlight the buckling pattern for 2D print/screen format. Quantified values of the microsphere curvature will not describe buckling wavelength, which is our main focus here. The buckling wavelengths reported were interpreted from the *spacing* of ripples on the surface renderings, i.e. the *spacing*

of the bands of high curvature. The values of r_curv in those bands might be related, in some way, to the amplitude of the buckling but this is not of less interest as we do not know, on a sphere-to-sphere basis, how far beyond the true critical buckling strain we are (as now mentioned in the paper, following reviewer 1's comments). We feel this makes any meaningful interpretation of r_curv difficult, and beyond the scope of this paper. We have added more discussion of the meaning of r_curv into the manuscript "These colour mappings are provided to highlight areas where the microsphere deviates from an ellipsoidal shape, i.e. where the shell has buckled." And "Values of rcurv at different strain levels or for different microsphere sizes are not provided here as the colour mappings (Figures 3–5, S1-5) are given simply to better highlight the buckling pattern".

7. A recent study on syntactic foam failure behaviors (European Journal of Mechanics-A/Solids, 2024, 105: 105256) aligns closely with the research theme and is recommended for appropriate citation.

We thank the reviewer for highlighting this impressive paper. Although it is focussed on hydrostatic loading, whereas we focus on uniaxial, it still provides excellent context for microstructure-property relations for microsphere composites. We have cited this paper several times in the introduction

8. This paper assumes all matrices have a Poisson's ratio of 0.49 (based on the incompressible assumption), while no experimental basis or justification is provided for this assumption.

The Poisson's ratio for elastomers is generally close to 0.5, and we have added a reference into the paper to support this. We have assumed 0.49 based some references based on experimental work on similar materials, which we have also referenced.
