# Supplementary material for: Buckling of elastomer-embedded thermoplastic microspheres under uniaxial compression revealed by in-situ X-ray micro-CT
Source: Commun Mater. 2026 Jun 5;7(1):236. doi: 10.1038/s43246-026-01214-w (PMC13561867; doi:10.1038/s43246-026-01214-w)
Supplement: Supplementary file 2 [file 43246_2026_1214_MOESM2_ESM.pdf]

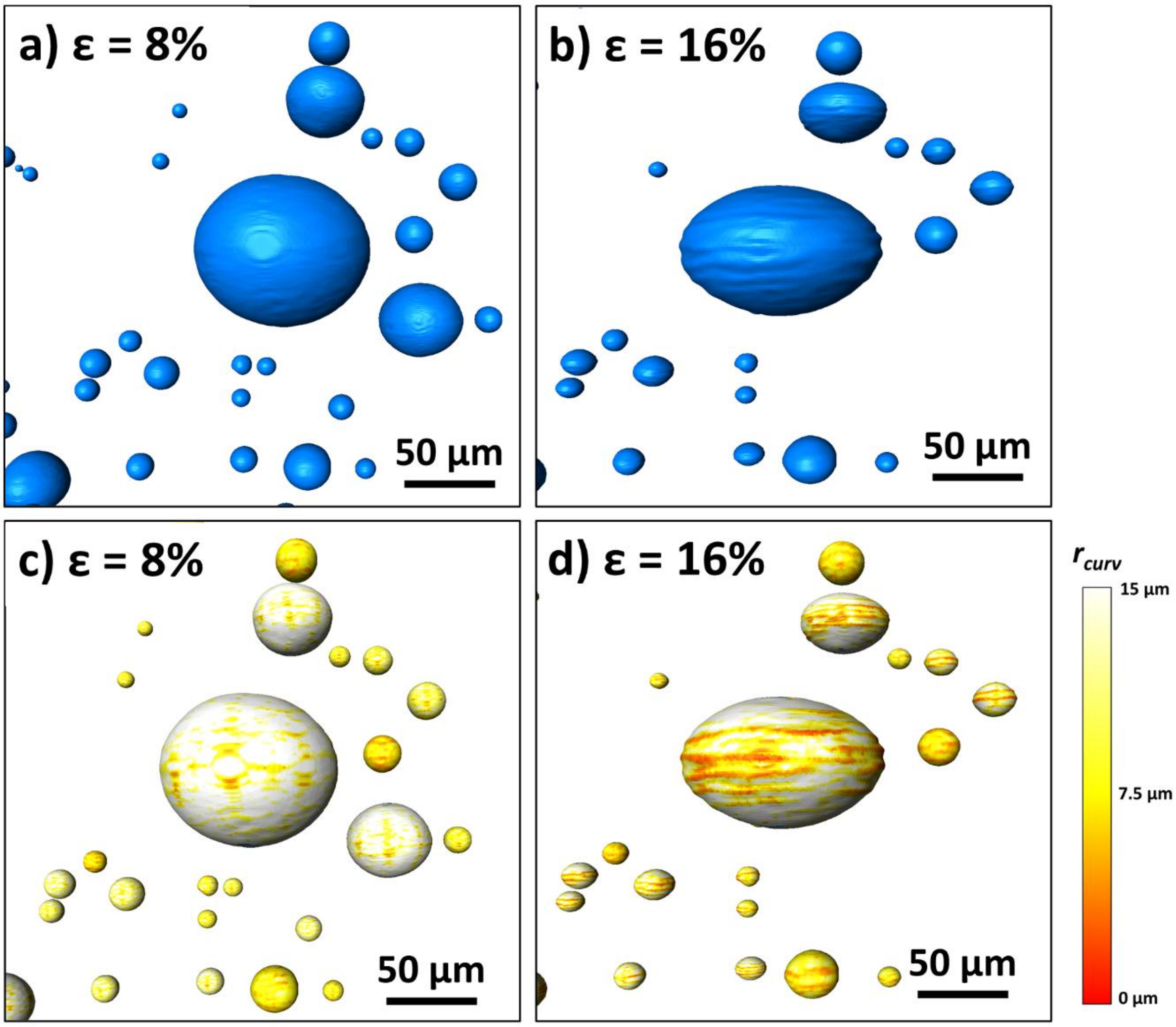

Figure S1 - Visualisation of Expancel 920 microspheres in ClearFlex95 before and after buckling*: a,b) surface renderings of the microspheres, showing rippling along the equator at the higher strains, c,d) surface renderings with radius-of-curvature colour overlay to further accentuate the equatorial buckling*

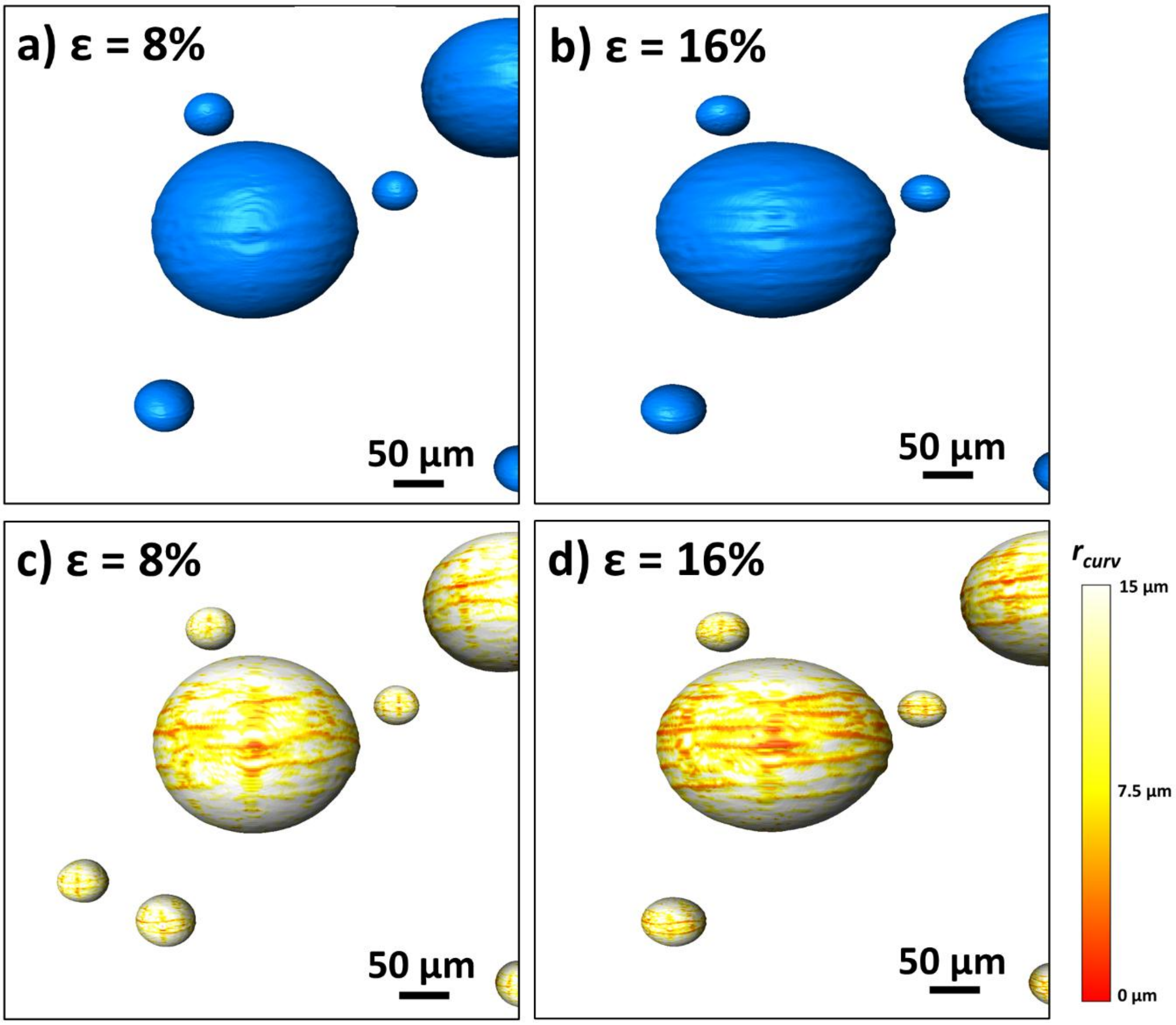

Figure S2 - Visualisation of Expancel 920 microspheres in Silicone before and after buckling: *a,b) surface renderings of the microspheres, showing rippling along the equator at the higher strains, c,d) surface renderings with radius-of-curvature colour overlay to further accentuate the equatorial buckling. Some evidence of buckling is visible here at the lower strain, which may be attributable to this sphere being particularly large (diameter = 205 µm) which, according to predictions from theory, should lead to a lower critical buckling wavelength (assuming an unchanged shell wall thickness).*

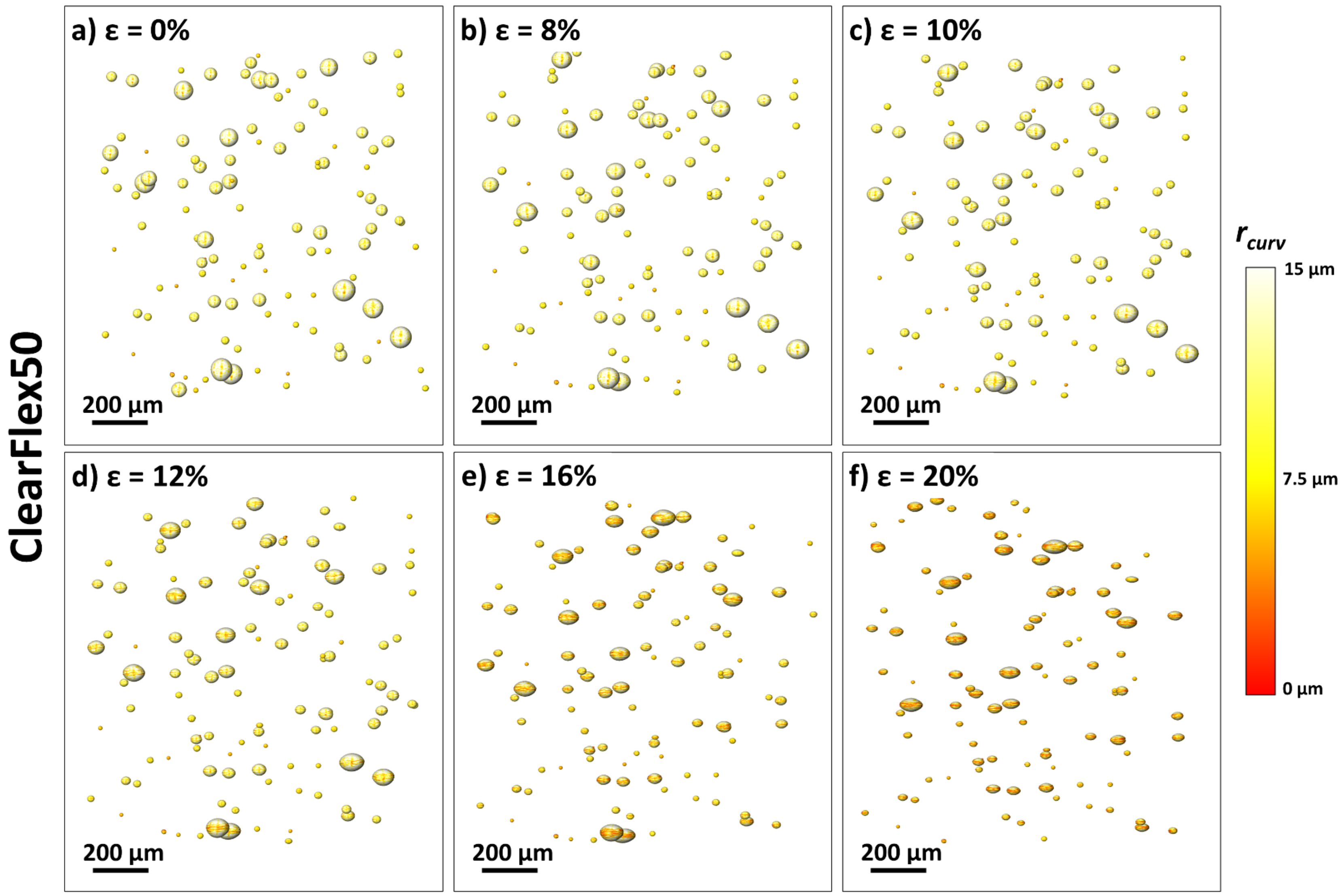

Figure S3- Expancel 920 microspheres in ClearFlex50 at a range of strains *a-f) Surface renderings of the segmented microspheres with added radius-of-curvature colourings to highlight where buckling has occurred.*

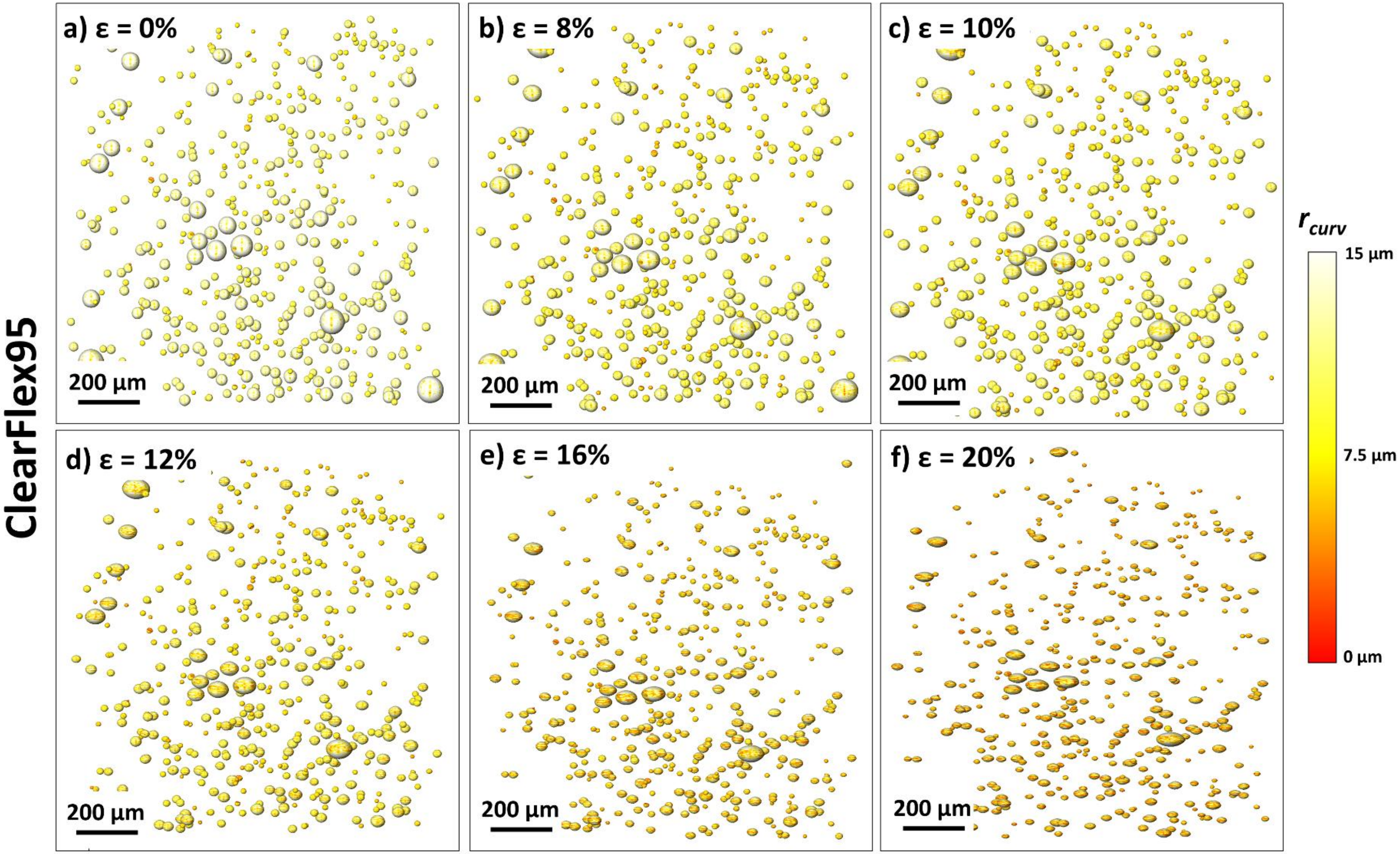

Figure S4 –920 microspheres in ClearFlex95 at a range of strains: *a-f) Surface renderings of the segmented microspheres with added radius-of-curvature colourings to highlight where buckling has occurred.*

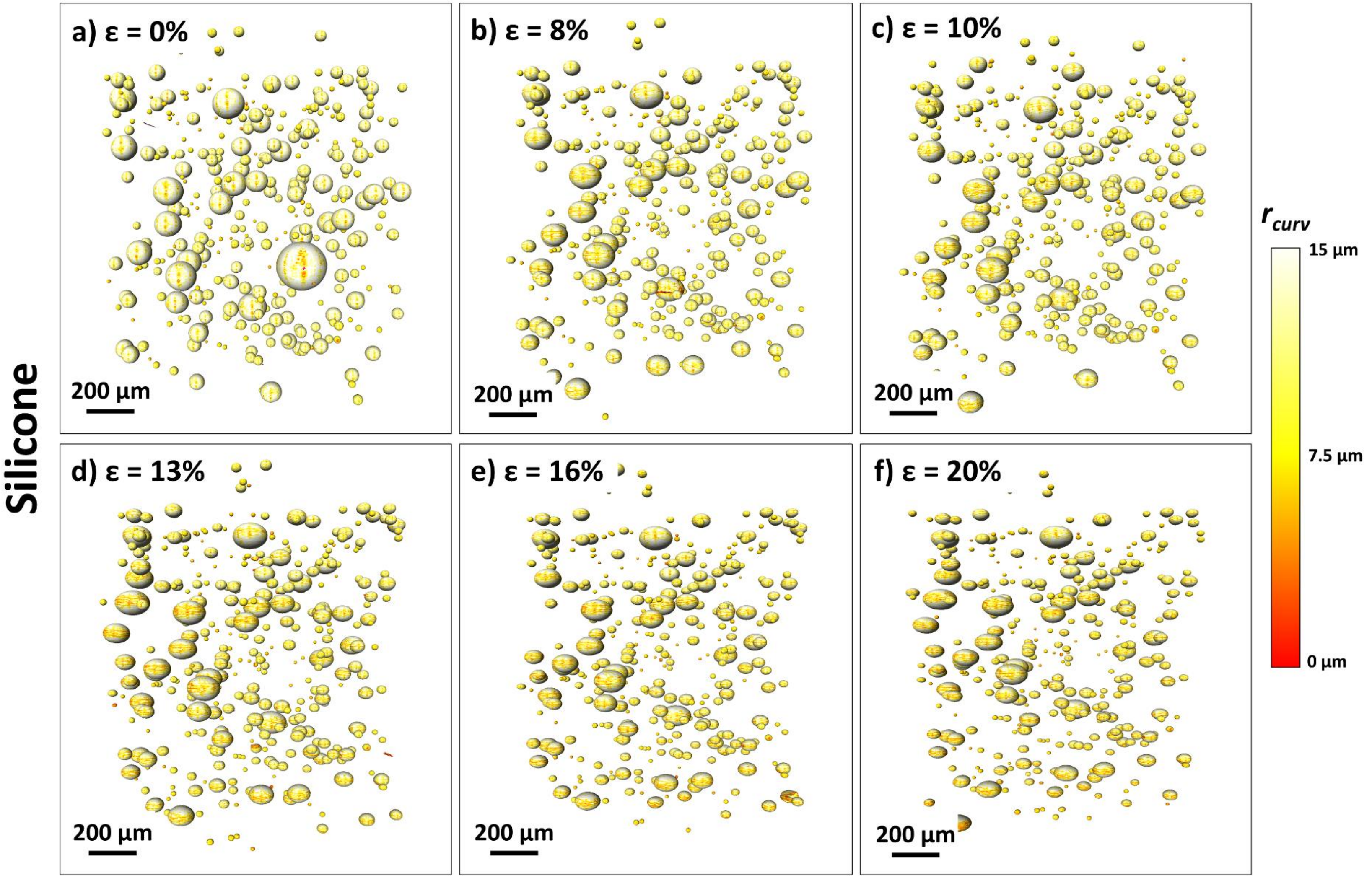

Figure S5 – Surface renderings of Expancel 920 microspheres in Silicone at a range of strains: *a-f) Surface renderings of the segmented microspheres with added radius-of-curvature colourings to highlight where buckling has occurred.*
